# 1-Benzyl-5-bromo-3-hydrazonoindolin-2-ones as Novel Anticancer Agents: Synthesis, Biological Evaluation and Molecular Modeling Insights

**DOI:** 10.3390/molecules28073203

**Published:** 2023-04-04

**Authors:** Tarfah Al-Warhi, Hadia Almahli, Raed M. Maklad, Zainab M. Elsayed, Mahmoud A. El Hassab, Ohoud J. Alotaibi, Nada Aljaeed, Rezk R. Ayyad, Hazem A. Ghabour, Wagdy M. Eldehna, Mohamed K. El-Ashrey

**Affiliations:** 1 Department of Chemistry, College of Science, Princess Nourah Bint Abdulrahman University, P.O. Box 84428, Riyadh 11671, Saudi Arabia; 2 Department of Chemistry, University of Cambridge, Cambridge CB2 1EW, UK; 3 Department of Pharmaceutical Chemistry, Faculty of Pharmacy, Kafrelsheikh University, Kafrelsheikh 33516, Egypt; 4 Scientific Research and Innovation Support Unit, Faculty of Pharmacy, Kafrelsheikh University, Kafrelsheikh 33516, Egypt; 5 Department of Medicinal Chemistry, Faculty of Pharmacy, King Salman International University (KSIU), South Sinai 46612, Egypt; 6 Department of Pharmaceutical Chemistry, Faculty of Pharmacy, Al-Azhar University, Cairo 11884, Egypt; 7 Department of Medicinal Chemistry, Faculty of Pharmacy, Mansoura University, Mansoura 35516, Egypt; 8 School of Biotechnology, Badr University in Cairo, Badr City 11829, Egypt; 9 Pharmaceutical Chemistry Department, Faculty of Pharmacy, Cairo University, Kasr Elini St., Cairo 11562, Egypt

**Keywords:** thiazole, anti-proliferative, isatin, MCF-7 cells, A-549 cells, VEGFR-2 inhibitor, molecular dynamics

## Abstract

Human health is experiencing several obstacles in the modern medical era, particularly cancer. As a result, the cancer therapeutic arsenal should be continually expanded with innovative small molecules that preferentially target tumour cells. In this study, we describe the development of two small molecule series (**7a**–**d** and **12a**–**e**) based on the 1-benzyl-5-bromoindolin-2-one scaffold that connected through a hydrazone linker to a 4-arylthiazole (**7a**–**d**) or 4-methyl-5-(aryldiazenyl)thiazole (**12a**–**e**) moiety. The anticancer activity of all the reported indolin-2-one derivatives was assessed against breast (MCF-7) and lung (A-549) cancer cell lines. The 4-arylthiazole-bearing derivatives **7c** and **7d** revealed the best anticancer activity toward MCF-7 cells (IC_50_ = 7.17 ± 0.94 and 2.93 ± 0.47, respectively). Furthermore, the VEGFR-2 inhibitory activity for **7c** and **7d** was evaluated. Both molecules disclosed good inhibitory activity, and their IC_50_ values were equal to 0.728 µM and 0.503 µM, respectively. Additionally, the impacts of **7d** on the cell cycle phases as well as on the levels of different apoptotic markers (caspase-3, caspase-9, Bax, and Bcl-2) were assessed. Molecular docking and dynamic simulations are carried out to explore the binding mode of **7d** within the VEGFR-2 active site.

## 1. Introduction

Numerous obstacles face human health in the modern medical era, particularly cancer [1]. Therefore, new small compounds should continuously be added to the treatment toolbox for cancer to specifically target tumour cells while posing little risk of damage to normal cells [2,3,4,5,6,7,8,9].

Angiogenesis is a complicated process that, under both physiological and pathological situations, is regulated by the creation of a wide variety of pro- and anti-angiogenic factors. Different diseases, such as rheumatoid arthritis, age-related macular degeneration, diabetic retinopathy, atherosclerosis, and cancer, have, however, been linked to an imbalance between these factors that govern angiogenesis [10]. Malignant cells must be substantially vascularized to get more oxygen and nutrients to survive and grow, making angiogenesis a crucial process. It was evidenced that the VEGF/VEGFR-2 signalling pathway stimulates several endothelial responses with a noticeable ability to increase the permeability of vessels and enhance the proliferation, differentiation, and migration of the cancerous endothelial cells [11,12]. As a result, the avoidance of angiogenesis through the suppression of VEGFR-2 could be a very effective cancer-fighting tactic [10].

**Figure 1:**
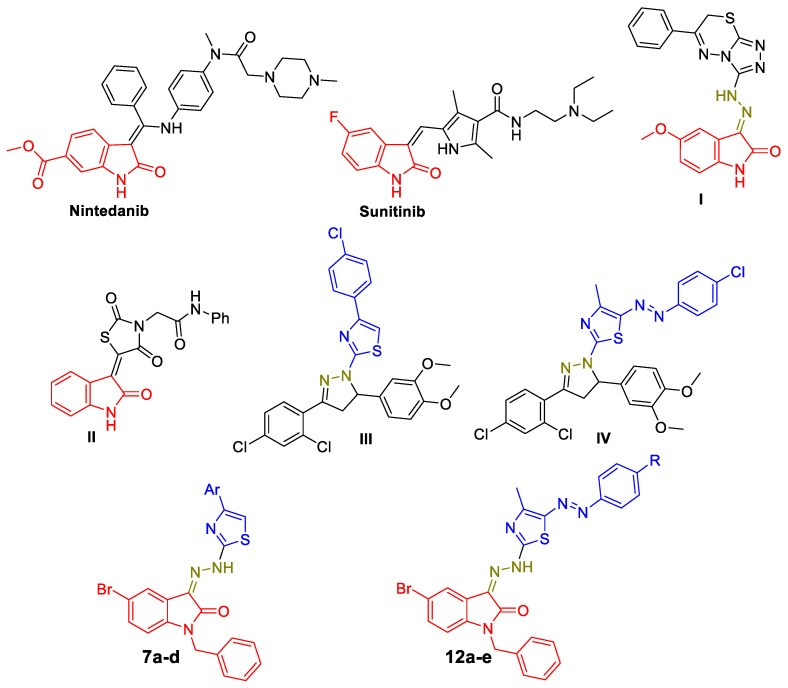
Structure of Nintedanib, Sunitinib, VEGFR-2 inhibitors (**I**–**IV**), and target molecules (**7a**–**d** and **12a**–**e**).

The isatin motif has surfaced as a privileged scaffold with numerous and significant pharmacological actions [13], such as antimicrobial [14], antiviral [15], and anticancer [16] effects. Noteworthy, isatin moiety has been included in some clinically approved medications such as Nintedanib and Sunitinib (Figure 1). As an important approach in drug design and discovery [17], several research studies have exploited the hybridization approach to develop diverse isatin-based conjugates as effective anticancer agents [18,19,20]. For instance, pyridine [21], azole [22], coumarin [23], thiazolo [3,2-*a*] benzimidazole [24], benzofuran [25,26], phthalazine [27], quinazoline [28], indole [29,30], thiazolidinone [31], and oxadiazole [32]. Additionally, it is worth mentioning that several studies have presented the isatin motif as a privileged scaffold for the development of diverse small molecule sets with significant kinase inhibitory activities [33].

After the FDA’s approval of the VEGFR-2 inhibitor Sunitinib, medicinal chemists have paid special attention to the identification of new isatin-based anti-cancer candidates targeting VEGFR-2 kinase [32,33,34,35,36,37,38,39,40,41,42,43,44]. Our research team has recently reported several VEGFR-2 inhibitors based on the isatin scaffold [45,46,47,48,49,50]. In 2020, we developed a new set of triazolo[3,4-*b*]thiadiazine—isatin hybrids with promising anti-angiogenic activities [46]. Compound **I** (Figure 1) disclosed broad antitumour activities in the NCI-USA anticancer assay (growth inhibition mean = 40%), along with good VEGFR-2 inhibition (IC_50_ = 435 nM). Moreover, the angiogenesis HET-CAM assay was used to check the mortality and toxicity of the target hybrids. Compound **I** remarkably reduced the developed blood vessels; in addition, it was not toxic for the chick embryos. In the last year, another small set of 2,4-thiazolidindione—isatin hybrids with promising anticancer and VEGFR-2 inhibitory activities were reported [47,48,49]. Compound **II** (Figure 1) revealed good anti-proliferative activities toward breast, lung, and colon cancer cell lines. Additionally, it exerted a potent inhibitory effect against VEGFR-2, with an IC_50_ of 84.05 nM.

On the other hand, another study has reported a series of pyrazoline derivatives decorated with 4-arylthiazole and 4-methyl-5-(aryldiazenyl)thiazole moieties as potent anticancer agents with an effective inhibitory effect against VEGFR-2. For example, compounds **III** and **IV** (Figure 1) displayed excellent cell growth inhibitory activity toward non-small lung cancer H441 and A549 cell lines, as well as good inhibitory activity against VEGFR-2 (IC_50_ = 185.5 nM and 113.3 nM, respectively) [50].

Utilizing the aforementioned findings, this article describes the development of two small molecule series (**7a**–**d** and **12a**–**e**) based on the 1-benzyl-5-bromoindolin-2-one scaffold that connected through a hydrazone linker to a 4-arylthiazole (**7a**–**d**) or 4-methyl-5-(aryldiazenyl)thiazole (**12a**–**e**) moiety. The anticancer activity for all the reported indolin-2-one derivatives was assessed against the VEGFR-2-overexpressing breast (MCF-7) and lung (A-549) cancer cell lines. Thereafter, the VEGFR-2 inhibitory activity was evaluated for the best anti-proliferative molecules. Moreover, the impact of target compounds on the cell cycle phases as well as on the levels of different apoptotic markers (caspases-3, caspase-9, Bax, and Bcl-2) was assessed. Molecular docking and dynamic simulations were carried out to explore the binding mode within the VEGFR-2 active site.

## 2. Results and Discussion

### 2.1. Chemistry

**Scheme 1:**
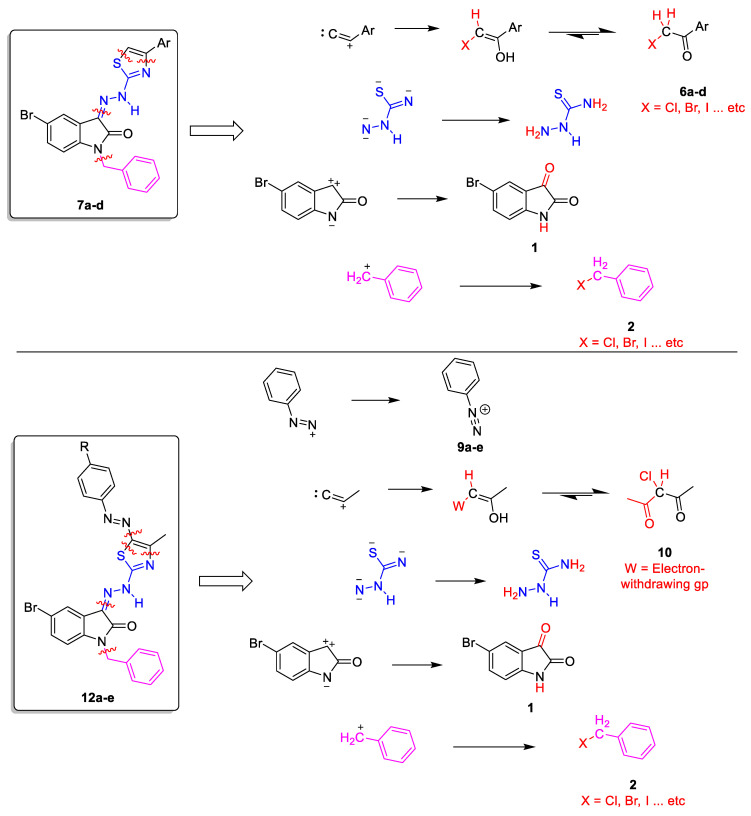
Retrosynthesis of target compounds **7a**–**d** and **12a**–**e**.

Target 1-benzyl-5-bromo-3-hydrazonoindolin-2-one derivatives **7a**–**d** and **12a**–**e** were subjected to retrosynthetic analysis to gain insights into potentially useful starting materials and chemical reagents to start their chemical syntheses (Scheme 1).

**Scheme 2:**
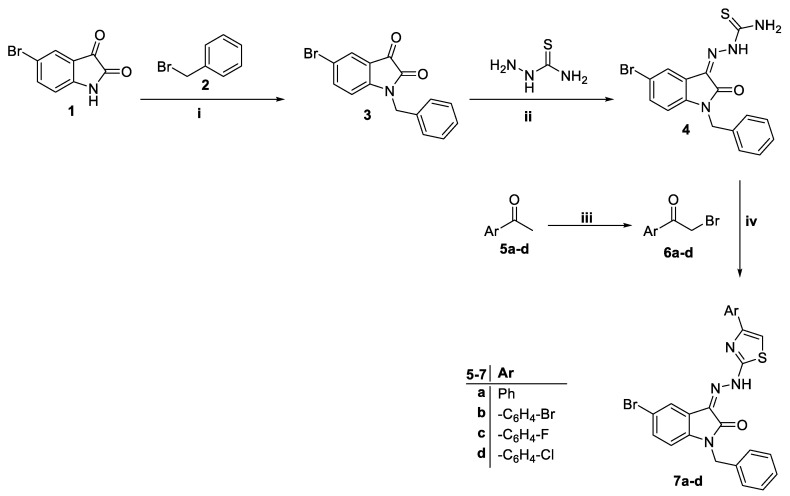
Reagents and conditions: (**i**) Anhydrous K_2_CO_3_, MeCN, r.t, 12 h; (**ii**) EtOH, cat. HOAc (a few drops), reflux 5 h; (**iii**) CuBr_2_ (2 equiv.), CHCl_3_/EtOAc 1:1, reflux 4 h; (**iv**) Absolute EtOH, reflux 7 h.

Upon applying the disconnection strategy to compounds **7a**–**d**, an aromatic *α*-haloketone (**6**), thiosemicarbazide, an isatin derivative (**1**), and benzyl halide (**2**) molecules were obtained. The retrosynthesis of target compounds **12a**–**e** was not remarkably different, except for the phenylazo moiety, which has inspired us with a diazonium salt (**9**), in addition to the aliphatic ketone (**10**) starting materials (Scheme 2).

Accordingly, *N*-benzylisatin derivative (**3**) was prepared by a nucleophilic substitution reaction of benzyl bromide (**2**) with isatin derivative (**1**) under S_N_2 reaction conditions, namely in a basic medium (anhydrous potassium carbonate) in a polar aprotic solvent (acetonitrile), as shown in Scheme 2. Then, the ketonic carbonyl group of compound (**3**) underwent a condensation reaction with thiosemicarbazide to furnish a thiosemicarbazone derivative (**4**, Scheme 2).

On the other hand, aromatic ketones (**5a**–**d**) were subjected to straightforward *α*-bromination using cupric bromide (Cu_2_Br) in a refluxing chloroform/ethyl acetate (1:1) solvent mixture following the reported method [51]. This afforded the ambident electrophilic reagents (**6a**–**d**) needed for the next step (Scheme 2) through a straightforward alternative route to the NBS-based [52,53] C-H bromination. Thereafter, the bifunctional electrophilic reagents (**6a**–**d**) were allowed to react with the ambident nucleophilic thiosemicarbazone substrate (**4**), affording the targeted thiazol-2-ylhydrazineylideneisatin derivatives **7a**–**d** (Scheme 2). The reaction involved regioselective [3+2] 4-substitutedthiazole ring cyclization in which the softer electrophilic motif (*α*-bromo Csp3) preferentially reacts with the softer nucleophilic one (sulfur) and the harder electrophilic one (ketonic carbonyl group) selectively reacts with the harder nucleophilic one (NH_2_). Although the *N*-alkylation reactions of nitrogen-based nucleophiles with *α*-halo carbonyl compounds are common [54,55], the issue discussed herein only represents a matter of competition between sulfur- and nitrogen-based nucleophilic sites for the electrophile. Analogous regioselectivity of similar cyclization reactions [22] and theoretical aspects [56] of the Hard and Soft Acid and Base Theory (HSABT) have been previously discussed elsewhere. It is of special interest to mention the ease with which the thiazole cyclization reaction took place even with neither acid nor base catalysis. This may be due to the high reactivity of the ketonic C=O (in series **6**) as compared to the relatively less reactive C=O of the ester analogues in similar reactions (which impose base catalysis, e.g., with NaOAc).

**Scheme 3:**
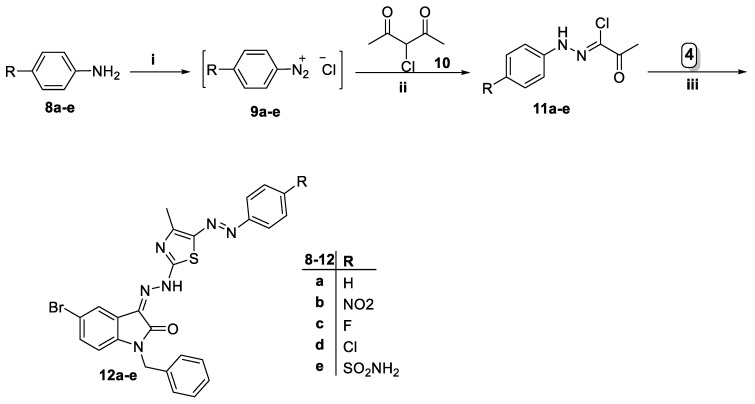
Reagents and conditions: (**i**) HCl 37% (2.5 equiv.), NaNO_2_ (1.1 equiv. added dropwise), dist. water, ice bath 0 °C, 30 min; (**ii**) NaOAc, EtOH/water, ice bath 0 °C, 2 h; (**iii**) Dioxane, TEA, reflux 5 h.

Analogously, target compounds **12a**–**e** (Scheme 3) were best obtained by considering the reagents and starting substrates inspired by their retrosynthetic analysis (shown in Scheme 1). Firstly, diazotization of anilines (**8a**–**e**) at 0 °C afforded the desired diazonium salts (**9a**–**e**), which were utilised as aqueous solutions in the next step. As for the azo-coupling reaction, it is considered a highly useful synthetic route to biologically active molecules [57]. More specifically, there is a possible disconnection route for the preparation of target compounds (**12a**–**e**) through azo-coupling of diazonium salts (e.g., **9a–e**) with thiazole scaffolds, which are prepared by reacting thiosemicarbazones (e.g., **4**) with 3-chloroacetylacetone (**10**) [58], where the coupling reaction took place at thiazole C5 [59]. Nevertheless, the current study considered the Japp-Klingemann pathway, which utilised a more favoured azo-coupling with an enolate ion.

Accordingly, the enolate anion of 3-chloroacetylacetone (prepared in situ by treatment of the latter with sodium acetate) was subjected to nucleophilic addition to the diazonium salt series (**9a**–**e**) via the Japp-Klingemann reaction [60] to afford the corresponding hydrazonoyl chlorides **11a**–**e** (Scheme 3).

After that, target 5-(phenylazo)thiazol-2-ylhydrazineylideneisatin derivatives (**12a**–**e**) were finally obtained by regioselective [3+2] thiazole ring cyclization, which was closely related to what has been discussed earlier regarding the last step in Scheme 2.

Although similar reactions were reported involving thiosemicarbazones derived from aromatic aldehydes or ketones, the analogous isatin-3-ylidene thiosemicarbazones were rarely studied. To the best of our knowledge, this is the second research work addressing the isatin-3-ylidene thiosemicarbazone motif reacting with hydrazonoyl chlorides obtained via the Japp-Klingemann pathway [61].

**Scheme 4:**
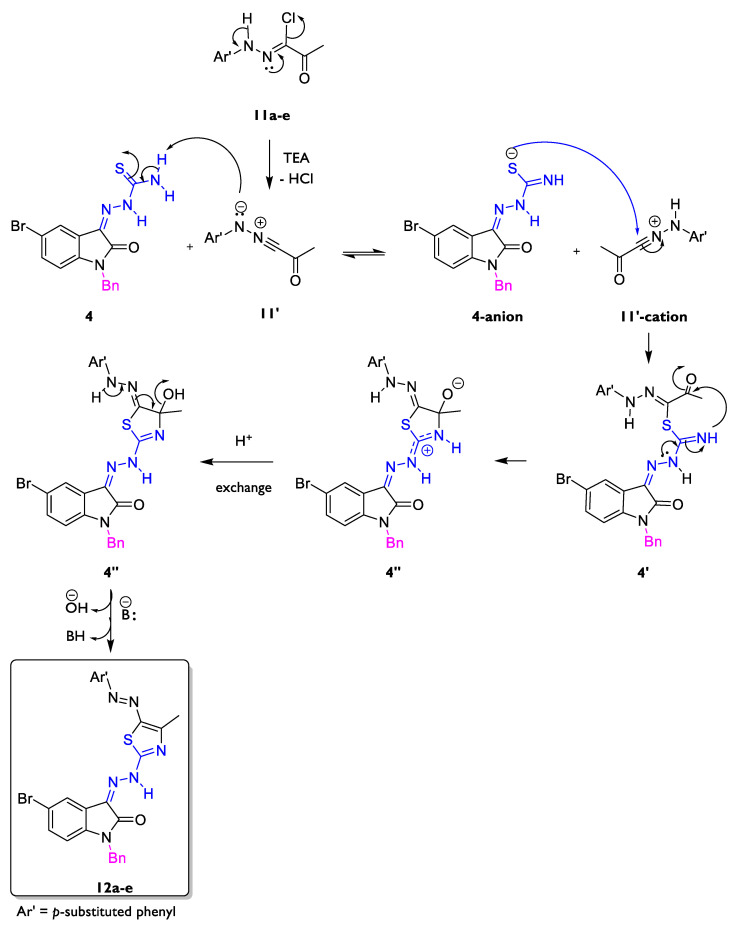
Suggested mechanism of the base-catalyzed 4-substituted thiazole cyclization reaction of hydrazonoyl chlorides (**11a**–**e**) with thiosemicarbazone (**4**) to afford target compounds (**12a**–**e**).

Furthermore, the reaction mechanism deserves much consideration, both in terms of the electrophilic species and the final cyclization step. Although the detailed evidence-based mechanistic study is beyond the scope of this article, it is noteworthy for reporting some relevant and controversial issues. In a few studies, it is suggested that the first step is a nucleophilic substitution of halide by sulphur (mostly by addition–elimination to C=N) [62,63]. This is while an alternative possibility of a nitrilimine intermediate (e.g., **11′**, Scheme 4) (whose existence was supported by X-ray-assisted DFT study alongside other experimental evidence in cycloaddition reactions and those involving hydrazonoyl chlorides in basic media [64,65]) still stands as a challenging one in the thiazole cyclization discussed herein (Scheme 4). Accordingly, the existence of nitrilimine (**11′**) was safely suggested herein upon the action of triethylamine on hydrazonoyl chlorides (**11a–e**) as in Scheme 4.

Furthermore, an acid-base equilibrium was suggested to take place in which the nitrilimine (**11′**) would act as a base that abstracts the relatively acidic NH proton of thiosemicarbazone (**4**). This equilibrium furnishes considerable concentrations of the activated electrophilic nitrilium-ion form of the former (**11′-cation**) alongside the activated nucleophilic anion form of the latter (**4-anion**), as outlined in Scheme 4. These equilibrium concentrations would be fairly sufficient for the next nucleophilic addition of the **4-anion** to the C-N triple bond of the nitrilium ion (**11′-cation**) to produce the open-chain hydrazonoyl thiolate intermediate (**4′**). Further intramolecular nucleophilic cycloaddition and subsequent tautomerism would afford the thiazolidine derivative (**4′′**), which would finally be driven to approach aromaticity by elimination of the water molecule to afford the desired thiazole derivatives (**12a**–**e**), see Scheme 4.

Finally, the chemical structures of target compounds **7a**–**d** and **12a**–**e** were confirmed by NMR spectroscopic analyses. The coexistence of the aromatic proton signals relevant to the isatin-3-ylidene, benzyl, and 4-methylthiazolyl moieties, showing the proper integrals for each, afforded strong evidence confirming the series **7a**–**d**. Additional aromatic proton signals of the phenylazo moiety showing the expected integral values provided evidence for confirming the structures of the series **12a**–**e**. It is noteworthy that according to the 1H NMR spectra, the target *N*-benzyl-5-bromoindolin-2-one derivatives exist as E- and Z-isomers. However, they interconvert rapidly in solution at room temperature and cannot be separated. Generally, the ratios of such indolinone hydrazone isomers are solvent- and temperature-dependent, as previously reported [66,67,68]. Thus, these isomers are not separable. We have reported several isatin hydrazone series that afford an E/Z mixture [25,27,69,70]. Further details are available in the “Experimental” section.

### 2.2. Biological Evaluation

#### 2.2.1. In Vitro Anti-Proliferative Activity

The anti-proliferative effect of the newly prepared *N*-benzyl-5-bromoindolin-2-one derivatives **7a**–**d** and **12a**–**e** was assessed in two human tumour cell lines: A-549 lung cancer and MCF-7 breast cancer. As a reference antitumour drug, doxorubicin was exploited in this experiment. The findings were expressed as median growth inhibitory concentration (IC_50_) values (Table 1).

As shown in Table 1, it is clear that a number of the prepared molecules had growth-inhibitory properties that ranged from good to modest for the examined tumour cell lines. Investigations into the anti-proliferative activity revealed that the MCF-7 cell line was more sensitive than the A-549 cell line to the effects of both series (**7a**–**d** and **12a**–**e**).

Concerning the growth inhibitory activity against MCF-7 cells, it is worth mentioning that the 4-arylthiazole bearing derivatives (**7a**–**d**) possessed better activity than their 4-methyl-5-(aryldiazenyl)thiazole analogous (**12a**–**e**). In particular, the 4-(*p*-chlorophenyl)thiazole bearing derivative **7d** (IC_50_ = 2.93 ± 0.47 µM) was the most potent counterpart with more enhanced activity than the reference drug doxorubicin (IC_50_ = 4.30 ± 0.84 µM). In addition, the 4-(*p*-fluorophenyl)thiazole bearing molecule **7c** showed good potency against MCF-7 cells (IC_50_ = 7.17 ± 0.94 µM). Furthermore, *N*-benzyl-5-bromoindolin-2-ones **7a** and **12d** were moderately potent toward MCF-7 cells, with IC_50_ values of 19.53 ± 1.05 and 13.92 ± 1.21 µM, respectively. This is while compounds **12a** and **12c** exhibited weak anticancer potency towards MCF-7 cells (IC_50_ = 39.53 ± 2.02 and 27.65 ± 2.39 µM, respectively).

On the other hand, evaluation of the anticancer potency in A-549 cells revealed that both series did not display significant anti-proliferative activity, except compounds **7d** and **12c**, which showed moderate impact with IC_50_ = 9.57 ± 0.62 and 12.20 ± 1.54, respectively.

It is worth stressing that the decoration of the pendant phenyl motif in series **7** with a small (fluoride) or moderate (chloride) lipophilic substituent enhanced the anticancer potency towards MCF-7 cells. In detail, the *para*-substituted derivatives **7c-d** disclosed 2.7- and 5.6-fold more enhanced activity (IC_50_ = 7.17 ± 0.94 and 2.93 ± 0.47 µM, respectively) than their un-substituted analogue **7a** (IC_50_ = 19.53 ± 1.05 µM). On the contrary, grafting a large lipophilic substituent such as 4-Br into compound **7b** diminished the activity.

In a similar vein, the substitution of the pendant phenyl moiety in series **12** with 4-F or 4-Cl (compounds **12c** and **12d**; IC_50_ = 27.65 ± 2.39 and 13.92 ± 1.21 µM, respectively) improved the growth inhibitory potency towards lung A-549 cells concerning the unsubstituted derivative **12a** (IC_50_ = 39.53 ± 2.02 µM).

Furthermore, in this study, we assessed the potential cytotoxic effect of the best anti-proliferative *N*-benzyl-5-bromoindolin-2-one derivatives **7c** and **7d** on the non-tumourigenic breast MCF-10A cells. The results disclosed that both indolin-2-ones **7c** and **7d** have modest cytotoxic impacts against the examined normal cells, with IC50 values of 91.33 ± 3.51 µM and 83.04 ± 4.10 µM, respectively.

#### 2.2.2. VEGFR-2 Inhibition

In this work, the most powerful cytotoxic molecules towards the MCF-7 cell line (**7c** and **7d**) were chosen to be tested for their VEGFR-2 inhibitory function. The VEGFR-2 inhibitor sorafenib, which has been successfully approved, served as the study’s reference medication. The findings were presented in the form of IC_50_ values, which can be found in Table 1. According to the findings, the molecules **7c** and **7d** that were investigated possessed a level of VEGFR-2 inhibitory activity that was moderate. Their IC_50_ values were equal to 0.728 µM and 0.503 µM, respectively (Table 2).

#### 2.2.3. Apoptosis Induction

The ability of cancer cells to stop apoptosis is directly linked to their ability to grow out of control. Thus, targeting apoptosis induction is an effective approach for preventing the growth of cancer cells [71,72]. In this study, we evaluated the apoptotic impact of **7d** as it proved to be the most active compound against the cell line MCF-7 (IC_50_ = 2.93 ± 0.47 µM) to investigate the effect of our compounds on the induction of the apoptotic cascade.

##### Effects on Activation of Proteolytic Caspases Cascade

The crucial apoptosis mediators are a family of cysteine-aspartic proteases known as caspases, whose activation has a significant impact on apoptosis induction [73]. Accordingly, the impact of 1-benzyl-5-bromoindolin-2-one derivative **7d** on caspase 3 was evaluated. Being the executioner caspase, its induction gives insight into the pro-apoptotic effect of **7d**. Moreover, the effect of **7d** on the activation of caspase-9 was determined for further evaluation of the pro-apoptotic activity.

Our results fathomed that **7d** induced the level of active caspase-3 by 18.3 fold as compared to the control, which implies its possession of significant pro-apoptotic activity. Furthermore, **7d** potentiated the level of active caspase-9 by 16.7 fold in comparison to the control. This implies that the intrinsic pathway was definitely adopted (Table 3).

##### Effects on Mitochondrial Apoptosis Pathway (Bcl-2 Family) Proteins

It is well established that the apoptotic switch on/off mechanism is precisely tuned by the Bcl-2 family of proteins, which are classified mainly into two classes: anti-apoptotic proteins such as Bcl-2 protein and the counteracting pro-apoptotic proteins, including Bax protein [74]. Inhibition of class I proteins and/or the activation of class II proteins can successfully induce apoptosis. Using Bcl-2 as a representative member of class I and Bax as a representative member of class II, we assessed the impact of **7d** on their levels in the MCF-7 cells. Moreover, the ratio between both proteins was detected as a determining factor for cell death regulation (Table 3).

As revealed in Table 3, **7d** downregulated the level of Bcl-2 by 4.9 folds in comparison to the control, while it effectively boosted the Bax level by 79.2 folds as compared to the control. The Bax/Bcl-2 ratio is a rather more indicative and conclusive value that precisely estimates the apoptotic effect. Notably, **7d** boosted this ratio by 159.56 folds as compared to the control. Collectively, the upregulation of Bax, the increase in the ratio of Bax to Bcl-2, and the downregulation of Bcl-2 all pointed to the suggestion that compound **7d** has a pro-apoptotic effect.

#### 2.2.4. Cell Cycle Analysis

**Figure 2:**
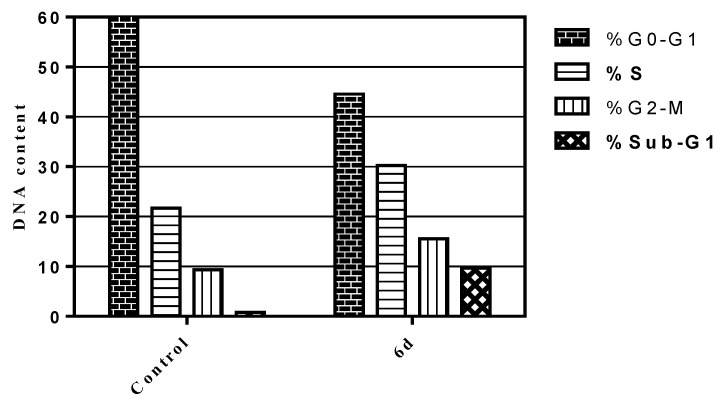
Effect of **7d** on the cell cycle phases within MCF-7 cells after treatment for 24 h at the IC_50_ concentration.

Cytotoxic agents abort cell growth by arresting their proliferation within definite, well-known checkpoints [75,76]. After cancerous cells were treated with anti-tumour agents, distinguishing cells in different cell cycle phases could be detected. MCF-7 cells have been treated with 1-benzyl-5-bromo-3-hydrazonoindolin-2-one **7d** at the IC_50_. Analyzing the gathered data reveals that compound **7d** significantly increased the number of cells in the sub-G1 phase compared to the control (9.7% vs. 0.8). It also resulted in a cell cycle arrest at the G2/M phase when compared to untreated cells (15.5% vs. 9.3%; Figure 2).

### 2.3. Molecular Modeling

#### 2.3.1. Docking Studies

In structural molecular biology and computer-assisted drug design, molecular docking is an essential technique. The objective of ligand–protein docking is to determine the most probable binding mode(s) between a ligand and a protein whose three-dimensional structure is known [77]. In this study, the 4ASD PDB file was used to perform the docking study of our newly synthesised molecule **7d** on the VEGFR2 active site. The reference drug Sorafenib was used as a guide to identifying the active binding site with the important key amino acid residues.

**Figure 3:**
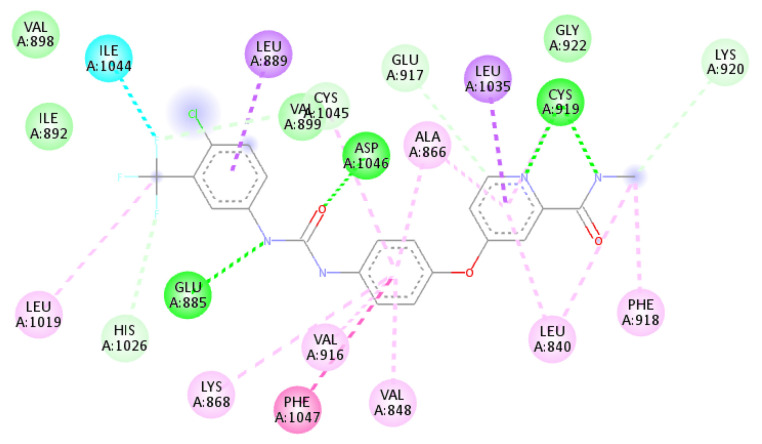
2D interaction of sorafenib with the VEGFR2 active binding site.

**Figure 4:**
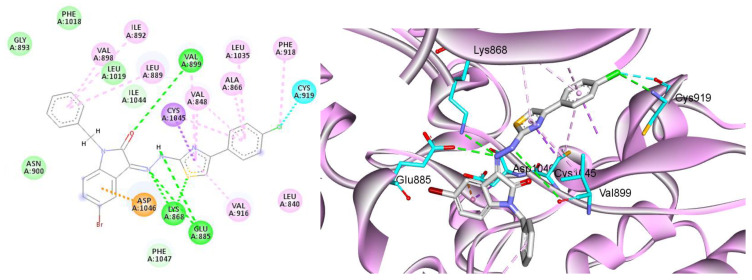
2D and 3D interactions of compound **7d** with the VEGFR2 active binding site.

Sorafenib shows a binding energy score of −16.4 kcal/mol and forms hydrogen bonding interactions with Glu885, Cys919, and Asp1046 amino acid residues through its nitrogen atoms and carbonyl oxygen (Figure 3). Compound **7d** showed a comparable good binding score of −15.6 kcal/mol. It also has a similar binding pattern, as it binds to Glu885 through hydrogen bonding with N, Asp106 through arene-H interaction, and Cys919 through halogen bonding with chlorine. These essential binding forces were supported by more interactions with Lys868, Val899 (hydrogen bonding), and several hydrophobic bindings with the aryl moieties (Figure 4).

#### 2.3.2. Molecular Dynamics

**Figure 5:**
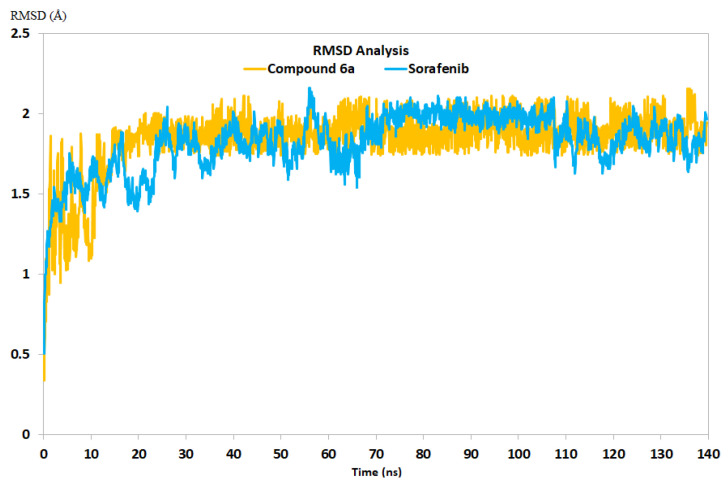
The RMSD values for sorafenib-VEGFR-2 (blue) and compound **7d**-VEGFR-2 (orange).

In several computational and molecular modelling investigations, molecular dynamics (MD) offers numerous crucial parameters, data, and figures. The exact assessment of the strength of the binding between a ligand and its target is one of the MD’s most often used applications. The composition of macromolecules and characterizing the impact of certain mutations on the resistance profile of various medications are two more well-reported uses [78]. As a result, it made logistical sense to use the MD’s advantages to support the biological and docking results for compound **7d**. Two MD simulation experiments were applied to compound **7d** bound to VEGFR-2 in addition to the x-ray coordinates of Sorafenib bound to VEGFR-2. It is interesting to note that the computed RMSD for compound **7d** reached 1.9 Å, while the RMSD of the Sorafenib complex reached 1.85 Å at their average deviations (Figure 5). The fact that compound **7d** can produce similar RMSD values to those of Sorafenib is a strong sign of its ability to form a stable complex with VEGFR-2.

**Figure 6:**
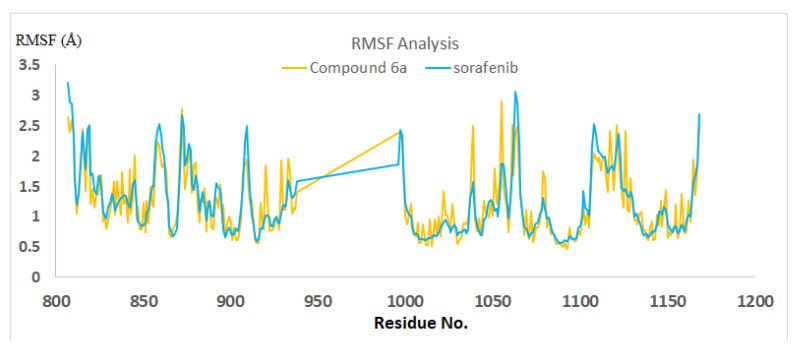
The RMSD values for sorafenib- VEGFR-2 (blue) and compound **7d**-VEGFR-2 (orange).

As expected, the acquired findings from the RMSF computations for each of the two complexes’ residues were very similar to the RMSD values. In comparison to the co-crystallized Sorafenib-VEGFR-2 complex, the compound **7d**-VEGFR-2 complex had a similar result in the residues fluctuation (Figure 6). The residue of VEGFR-2 after the binding of compound **7d** had average RMSF values of 1.52 Å while sorafenib induced average RMSF values of 1.47 Å. Given the ability of compound **7d** to produce similar values of RMSD and RMSF to Sorafenib, it is projected that VEGFR-2 is the main target for compound **7d**.

## 3. Conclusions

With the prime aim of developing novel anti-tumour agents, this study reported the design and synthesis of two small molecule series (**7a**–**d** and **12a**–**e**) based on the 1-benzyl-5-bromoindolin-2-one scaffold that connected through a hydrazone linker to a 4-arylthiazole (**7a**–**d**) or 4-methyl-5-(aryldiazenyl)thiazole (**12a**–**e**) moiety. The target molecules displayed cell growth inhibitory activities toward breast (MCF-7) and lung (A-549) cancer cell lines in variable degrees. In particular, the 4-arylthiazole bearing derivatives **7c** and **7d** revealed the best anticancer activity toward MCF-7 cells (IC_50_ = 7.17 ± 0.94 and 2.93 ± 0.47, respectively). Furthermore, both molecules disclosed good VEGFR-2 inhibitory activity, and their IC_50_ values were equal to 0.728 µM and 0.503 µM, respectively. Additionally, the impacts of **7d** on the cell cycle phases as well as on the levels of different apoptotic markers (caspase-3, caspase-9, Bax, and Bcl-2) were assessed. Collectively, the upregulation of Bax, the increase in the ratio of Bax to Bcl-2, and the downregulation of Bcl-2 all pointed to the suggestion that compound **7d** has a pro-apoptotic effect. Finally, molecular modelling studies have been carried out to rationalise the VEGFR-2 inhibitory activity of target compounds. The docking analysis disclosed the ability of 7d to achieve the essential interactions within the VEGFR-2 active site. Moreover, MD analysis revealed the ability of compound **7d** to produce similar values of RMSD and RMSF to Sorafenib.

## 4. Experimental

### 4.1. Chemistry

#### 4.1.1. General

The NMR spectra were recorded by a Bruker Avance III 400 MHz high-performance digital FT-NMR spectrophotometer. ^1^H and ^13^C spectra were run at 400 and 100 MHz, respectively, in deuterated dimethylsulphoxide (DMSO-*d*_6_). All coupling constant (*J*) values are given in hertz. Chemical shifts (*δ*_C_) are reported relative to DMSO-*d*_6_ as internal standards. IR spectra were recorded as potassium bromide discs on a Schimadzu FT-IR 8400S spectrophotometer.

*N*-Benzyl-5-bromoindoline-2,3-dione **3** [79], 2-bromo-1-arylethanones **6a**–**d** [80], and 2-oxo-*N*′-(4-substitutedphenyl) propanehydrazonoyl chloride **11a**–**e** [81] were prepared as reported earlier.

#### 4.1.2. 2-(1-Benzyl-5-bromo-2-oxoindolin-3-ylidene)hydrazine-1-carbothioamide 4

To a hot stirred solution of *N*-benzyl-5-bromoindoline-2,3-dione **3** (2.5 g, 8.0 mmol) in ethanol (15 mL) and cat. HOAc, thiosemicarbazide (0.9 g, 10 mmol) was added. Stirring then continued at the refluxing temperature for 5 h. The precipitate was filtered, washed with cooled ethanol, dried, and recrystallized from glacial acetic acid to afford the key intermediate **4** (yield 40%), m.p. 241-243 °C; ^1^H NMR (DMSO-*d*_6_) *δ ppm*: 4.956 (s, 2 H, benzylic protons), 6.94 (d, 1 H, Ar-H, *J* = 8.4 Hz), 7.25-7.35 (m, 5 H, Ar-H), 7.48 (dd, 1 H, Ar-H, *J* = 8.4, 2.0 Hz), 7.92 (d, 1 H, Ar-H, *J* = 2.0 Hz), 8.86 (s, 1 H, NH), 9.15 (s, 1 H, NH), 12.21 (s, 1 H, NH); ^13^C NMR (DMSO-*d*_6_) *δ ppm*: 43.04, 112.73, 115.43, 122.27, 123.79, 127.82, 127.88, 128.09, 129.15, 129.99, 133.41, 135.89, 142.00, 160.89, 179.23; Anal. Calcd. for C_16_H_13_BrN_4_OS: C, 49.37; H, 3.37; N, 14.39, found C, 49.22; H, 3.39; N, 14.43 [82,83].

#### 4.1.3. General Procedure for the Preparation of 1-benzyl-5-bromo-3-(2-(4-arylthiazol-2-yl)hydrazono)indolin-2-one **7a**–**d**

The key intermediate **4** (0.2 g, 0.5 mmol) was added to a solution of the appropriate 2-bromo-1-arylethanone **6a**–**d** (0.55 mmol) in ethanol (8 mL). The mixture was refluxed for seven hours. The precipitate was recovered by filtration while hot, washed with diethyl ether, dried, and recrystallized from methanol/DMF to provide the desired isatin derivatives **7a**–**d** with yields ranging from 69 to 81%.

#### 4.1.4. General Procedure for Preparation of 1-benzyl-5-bromo-3-(2-(4-methyl-5-(aryldiazenyl)thiazol-2-yl)hydrazono)indolin-2-ones **12a**–**e**

The key intermediate **4** (0.2 g, 0.5 mmol) was added to a solution of the appropriate hydrazonoyl chloride derivative **11a**–**e** (0.5 mmol) in dioxane (8 mL) in the presence of a catalytic amount of triethylamine. For 5 h, the reaction mixture was refluxed. The precipitate was recovered by filtering while hot, washed with ethanol, dried, and recrystallized from ethanol/DMF to obtain isatin derivatives **12a**–**e** with a yield of 58–70%.

### 4.2. Pharmacological Assays

The experimental procedures for the performed pharmacological assays. MTT cytotoxicity [84,85,86], VEGFR-2 inhibitory activity [87,88], and cell cycle flow cytometry [89] assays, as well as the assessment of the expression levels for the apoptotic markers [89], were described in the Supplementary Materials and performed as previously described.

### 4.3. Molecular Modeling Studies

#### 4.3.1. Docking Studies

The entire docking investigation was carried out using Vina Autodock software [90]. The VEGFR-2 crystal structure was acquired from the protein data bank using the PDB IDs: 4ASD. Chemdraw was used to draw a hybrid **7d**, which was then transformed into a 3D structure using the Biovia Discovery Visualizer. Because Vina Autodock requires the receptor and ligands to be in pdbqt format, MGL tools were developed to produce the necessary pdbqt files. Furthermore, the active site was built by constructing a grid box around the binding of the co-crystallised sorafenib with dimensions of 20, 20, and 20 in the X, Y, and Z axes, respectively. Finally, hybrid **7d** was docked onto the VEGFR-2 predetermined active site. The docking findings were displayed using the free Biovia Discovery Studio 2021 visualizer, which generated 2D and 3D interactions for the docked poses.

#### 4.3.2. Molecular Dynamics

Groningen Machine for Chemical Simulations (GROMACS) 5.1.1 software was used to run the necessary molecular dynamics (MD) simulations [91]. Two molecular dynamic simulations (MDS) were performed using the 3D coordinates of VEGFR-2 in a complex with sorafenib and hybrid **7d**. The topologies of hybrid **7d** and sorafenib were generated using the GlycoBioChem PRODRG2 Server under the GROMOS96 force field [92]. The protein topology was generated using the pdb2gmx function embedded in the GROMACS software and then joined with each ligand topology to produce two o protein-ligand complexes. A typical workflow of GROMACS simulations was applied to the two complexes, starting with solvation using the recommended single point charge (SPC) water model, followed by adding the suitable number of counter-ions to neutralise the two complexes [93,94,95,96]. Energy-minimised complexes were obtained by applying a maximum of 50,000 steps of steepest descent minimization under the GROMOS96 53A6 force field using a Cutoff less than 10.0 kJ/mol. As a core step, the two complexes were equilibrated to the desired temperature (310 K) and pressure (1 atm) using both NVT and NPT ensembles for 8 ns and 2 ns, respectively. The particle mesh Ewald (PME) technique with a 12 cut-off and 12 Fourier spacing was used to calculate the long-range electrostatic values. All the systems underwent a 150 ns production stage. The structural coordinates were stored every 30 ps, and every two subsequent steps were separated by 2 fs. Throughout the simulation, the temperature (310 K) and the pressure (1 atm) were controlled via the Parrinello-Rahman technique and the V-rescale weak coupling method (a modified Berendsen thermostat) [97,98]. The obtained trajectories from the production stage were used to determine the root means square deviation (RMSD) and root mean square fluctuation (RMSF) of the overall system and each residue, respectively.

## Figures and Tables

**Table 1:**
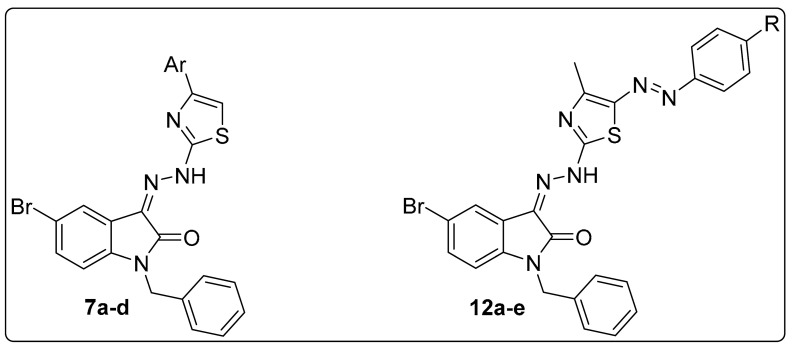
In vitro anticancer activities (IC_50_) for the synthesised *N*-benzyl-5-bromoindolin-2-one derivatives **7a**–**d** and **12a**–**e** against MCF-7 and A-549 cell lines.

Comd.	Ar/R	IC_50_ (µM) ^a^	
		MCF-7	A-549
**7a**	C_6_H_5_	19.53 ± 1.05	NA ^b^
**7b**	4-Br-C_6_H_4_	NA ^b^	NA ^b^
**7c**	4-F-C_6_H_4_	7.17 ± 0.94	20.43 ± 1.51
**7d**	4-Cl-C_6_H_4_	2.93 ± 0.47	9.57 ± 0.62
**12a**	H	39.53 ± 2.02	61.76 ± 4.01
**12b**	NO_2_	NA ^b^	NA ^b^
**12c**	F	27.65 ± 2.39	12.20 ± 1.54
**12d**	Cl	13.92 ± 1.21	29.45 ± 2.06
**12e**	SO_2_NH_2_	NA ^b^	NA ^b^
**Dox.**	-	4.30 ± 0.84	6.4 ± 0.79

^a^ IC_50_ values are presented as means ± S.D. for 3 separate experiments. ^b^ NA: IC_50_ higher than 100 µM.

**Table 2:** IC_50_ for the VEGFR-2 inhibitory action of **7c** and **7d**.

Compound	VEGFR-2 IC_50_ (μM) ^a^
**7c**	0.728
**7d**	0.503
**Sorafenib**	0.112

^a^ IC_50_ values are the mean ± SD of three separate experiments.

**Table 3:** Effect of *N*-benzylindolin-2-one derivative **7d** on the active caspases-3 and -9 levels and the Bax and Bcl-2 expression levels in MCF-7 cells after treatment with **7d** at its IC_50_ concentration.

Comp.	Caspase-3 IU/mL	Caspase-9 IU/mL	Bax IU/mL	Bcl-2 IU/mL	Bax/Bcl-2 Ratio
**Control**	0.0156	0.0111	1.3	3.137	0.4144
**7d**	0.2862 ***	0.1853 **	102.9 ***	0.6449 **	159.56

** Significantly different from control at *p* < 0.01. *** Significantly different from control at *p* < 0.001.

## Data Availability

Not applicable.

## Supplementary Materials

The following supporting information can be downloaded at: https://www.mdpi.com/article/10.3390/molecules28073203/s1, Spectra and Elemental data for target compounds.

## Sample Availability

Samples of the compounds **6** and **8** are available from the authors.
