# Supplementary material for: 1-Benzyl-5-bromo-3-hydrazonoindolin-2-ones as Novel Anticancer Agents: Synthesis, Biological Evaluation and Molecular Modeling Insights
Source: Molecules. 2023 Apr 4;28(7):3203. doi: 10.3390/molecules28073203 (PMC10096524; doi:10.3390/molecules28073203)
Supplement: Supplementary file 1 [file molecules-28-03203-s001.zip › molecules-2284919-supplementary.pdf]

Supporting Information

1-Benzyl-5-bromo-3-hydrazonoindolin-2-ones as novel anticancer agents: Synthesis, biological evaluation and molecular modeling insights

Tarfah Al-Warhi, Hadia Almahli, Raed M. Maklad, Zainab M. Elsayed, Mahmoud A. El Hassab, Ohoud J. Alotaibi, Nada Aljaeed, Rezk R. Ayyad, Hazem A. Ghabour, Wagdy M. Eldehna, Mohamed K. El-Ashrey

Tables of Contents

1. Anti-proliferative Activity 2

2. VEGFR-2 Kinase Inhibitory Activity 3

3. Cell Cycle Analysis 3

4. Spectra and Elemental data for target compounds 4-7

1. Anti-proliferative activity

The two examined human cancer cell lines (MCF-7 and A-549) have been obtained from American Type Culture Collection (ATCC). Cells lines were maintained as monolayers in Dulbecco's Modified Eagle's Medium (DMEM) supplemented with 10% FBS, 2 mM L-glutamine, 100 U/ml penicillin and 100µg/ml streptomycin sulfate. Cells were sub-cultured with trypsine /EDTA solution, counted with haemocytometer and plated onto 96-well plates (5000 cells/well) and left overnight to form a semi-confluent monolayer. Cell monolayers were treated in quadrates with vehicle (DMSO, 0.1% v/v), test samples (**7a-d** and **12a-e**) or doxorubicin as positive control for an exposure time of 48 h. At the end of exposure, MTT solution in PBS (5 mg/ml) was then added to all well including no cell blank and left to incubate for 90 min. The formation of formazan crystals were visually confirmed using phase contract microscopy. DMSO (100 µl/well) was added to dissolve the formazan crystals with shaking for 10 min after which the absorbance was read at 590 nm against no cell blanks on a FLuo Star Optima microplate reader (BMG technologies, Germany). Cell proliferation was calculated comparing the OD values of the DMSO control wells and those of the samples represented as % proliferation to the control. Dose-response experiment was performed on samples producing > or =50% loss of cell proliferation using five serial 2-fold dilutions (50, 25, 12.5, 6.25 and 3.125 µM) of the sample. IC_{50} values (concentration of sample causing 50% loss of cell proliferation of the vehicle control) were calculated using non-linear regression curve fitting of the dose response plots on GraphPad Prism V.6.0 software.

2. VEGFR-2 Kinase Assay

In vitro VEGFR-2 inhibitory activities of compounds **7c** and **7d** and Sorafenib were evaluated using serial dilutions (10, 5, 2.5, 1, 0.1, 0.01 μM) against VEGFR-2 Kinase Assay Kit Catalog # 40325 (BPS Bioscience) according to manufacturer's instructions. In brief, the master mixture was produced (25 μL per well) and poured into each well. Each well received 5 μL of inhibitor solution designated as "Test Inhibitor". The "Positive Control" and "Blank" groups received 5 μL of the same solution without the inhibitor (Inhibitor buffer). In order to prepare 3 mL of kinase buffer, 600 μL of kinase buffer were combined with 2400 μL of water. The blank wells received 20 μL of kinase buffer. The amount of VEGFR-2 required for the test was measured and the enzyme was diluted to 1 ng/ μL with kinase buffer. 20 μL of diluted VEGFR-2 enzyme was added to the wells designated as "Test Inhibitor Control" and "Positive Control" to start the reaction and the mixtures were incubated at 30 °C for 45 minutes. After the 45 minutes, each well received 50 μL of KinaseGlo Max reagent and the plate was incubated at room temperature for 15 minutes. The luminescence was measured with a microplate reader.

The autophosphorylation percentage inhibition by compounds was calculated using the following equation:

$$100\% - [(negative\ control)/(positive\ control) - (negative\ control)]$$

Using the curves of percentage inhibition of five concentrations of each compound, IC_{50} was calculated.

3. Cell Cycle Analysis

Breast cancer MCF-7 cells were treated with 1-benzyl-5-bromo-3-hydrazonoindolin-2-one **7d** for 24 h (at its IC_{50} concentration), and then cells were washed twice with ice-cold phosphate buffered saline (PBS). Subsequently, the treated cells were collected by centrifugation, fixed in ice-cold 70% (*v*/*v*) ethanol, washed with PBS, re-suspended with 100 μg/mL RNase, stained with 40 μg/mL PI, and analyzed by flow cytometry using FACS Calibur (Becton Dickinson, BD, Franklin Lakes, NJ, USA). The cell cycle distributions were calculated using CellQuest software 5.1 (Becton Dickinson).

4. Spectra and Elemental data for target compounds

2-(1-Benzyl-5-bromo-2-oxoindolin-3-ylidene)hydrazine-1-carbothioamide (4).

Yellow crystals (yield 78%), m.p. 248-250 °C; ^{1}H NMR (DMSO-d_6) δ *ppm*: 6.94 (d, 1 H, Ar-H, J = 8.4 Hz), 7.25-7.35 (m, 5 H, Ar-H), 7.48 (dd, 1 H, Ar-H, J = 8.4, 2.0 Hz), 7.92 (d, 1 H, Ar-H, J = 2.0 Hz), 8.86 (s, 1 H, NH), 9.15 (s, 1 H, NH), 12.21 (s, 1 H, NH); ^{13}C NMR (DMSO-d_6) δ *ppm*: 43.04, 112.73, 115.43, 122.27, 123.79, 127.82, 127.88, 128.09, 129.15, 129.99, 133.41, 135.89, 142.00, 160.89, 179.23; Anal. Calcd. for $C_{16}H_{13}BrN_4OS$: C, 49.37; H, 3.37; N, 14.39, found C, 49.22; H, 3.39; N, 14.43.

1-Benzyl-5-bromo-3-(2-(4-phenylthiazol-2-yl)hydrazono)indolin-2-one (7a).

Orange powder (yield 84%), m.p. 223-225 °C; IR (KBr, ν cm^{-1}): 3197 (C=O) and 1664 (NH); ^{1}H NMR (DMSO-d_6) δ *ppm*: 4.96, 5.03 (2s, 2H, CH_2), 6.89 (d, 0.5 H, Ar-H, J = 8.4 Hz), 7.02 (d, 0.5 H, Ar-H, J = 8.4 Hz), 7.27-7.51 (m, 9.5 H, Ar-H), 7.69 (d, 1 H, Ar-H, J = 5.8 Hz), 7.83 (d, 1 H, Ar-H, J = 8.4 Hz), 7.90 (d, 1 H, Ar-H, J = 8.4 Hz), 8.56 (s, 0.5 H, Ar-H), 13.06, 13.24 (2s, 1H, NH); ^{13}C NMR (DMSO-d_6) δ *ppm*: 169.70, 161.40, 148.06, 143.23, 138.18, 136.01, 135.32, 132.33, 129.21, 128.84, 128.51, 127.87, 127.05, 127.63, 126.87, 123.79, 121.44, 119.68, 117.35, 111.07, 43.21; MS *m/z* [%]: 489.01 [M^{+}, 3.12]; Anal. Calcd. for $C_{24}H_{17}BrN_4OS$: C, 58.90; H, 3.50; N, 11.45, found C, 59.11; H, 3.48; N, 11.37.

1-Benzyl-5-bromo-3-(2-(4-(4-bromophenyl)thiazol-2-yl)hydrazono)indolin-2-one (7b).

Orange powder (yield 76%), m.p. 248-249 °C; IR (KBr, ν cm^{-1}): 1662 (C=O) and 3062 (NH); ^{1}H NMR (DMSO-d_6) δ *ppm*: 4.94, 5.01 (2s, 2H, CH_2), 6.88 (d, 0.5 H, Ar-H, J = 8.4 Hz), 6.99 (d, 0.5 H, Ar-H, J = 8.4 Hz), 7.24-7.42 (m, 8 H, Ar-H), 7.48 (dd, 0.5 H, Ar-H, J = 8.4, 1.6 Hz), 7.59 (d, 0.5 H, Ar-H, J = 8.4 Hz), 7.67-7.85 (m, 2.5 H, Ar-H), 8.55 (s, 0.5 H, Ar-H), 12.21, 13.21 (2s, 1H, NH, D_2O exchangeable); ^{13}C NMR (DMSO-d_6) δ *ppm*: 56.47, 108.82, 112.77, 115.45, 121.57, 121.84, 122.41, 127.64, 127.82, 128.14, 128.18, 129.12, 129.18, 132.11, 132.28, 132.87, 136.00, 140.97, 161.36; Anal. Calcd. for $C_{24}H_{16}Br_2N_4OS$: C, 50.73; H, 2.84; N, 9.86, found C, 50.90; H, 2.82; N, 9.92.

1-Benzyl-5-bromo-3-(2-(4-(4-fluorophenyl)thiazol-2-yl)hydrazono)indolin-2-one (7c).

Orange powder (yield 75%), m.p. 230-231°C; IR (KBr, ν cm^{-1}): 1701 (C=O) and 3047

(NH); ^{1}H NMR (DMSO-d_6) δ *ppm*: 4.94, 5.01 (2s, 2H, CH_2), 6.88 (d, 0.5 H, Ar-H, J = 8.4 Hz), 7.00 (d, 0.5 H, Ar-H, J = 8.4 Hz), 7.23-7.42 (m, 8 H, Ar-H), 7.48 (dd, 0.5 H, Ar-H, J = 8.4, 2.0 Hz), 7.65 (s, 0.5 H, Ar-H), 7.67 (s, 0.5 H, Ar-H), 7.85-7.95 (m, 2 H, Ar-H), 8.55 (s, 0.5 H, Ar-H), 13.15, 13.22 (s, 1H, NH, D_2O exchangeable); ^{13}C NMR (DMSO-d_6) δ *ppm*: 56.74, 107.73, 112.76, 114.41, 115.43, 115.94, 116.15, 116.42, 121.86, 122.38, 127.64, 127.82, 128.11, 128.21, 128.28, 128.52, 129.12, 129.18, 130.26, 132.83, 136.01, 136.91, 140.95, 161.13, 161.37; MS *m/z* [%]: 506.90 [M^+, 9.75]; Anal. Calcd. for $C_{24}H_{16}BrFN_4OS$: C, 56.81; H, 3.18; N, 11.04, found C, 56.68; H, 3.20; N, 11.12.

1-Benzyl-5-bromo-3-(2-(4-(4-chlorophenyl)thiazol-2-yl)hydrazono)indolin-2-one (7d). Yellow powder (yield 83%), m.p. 242-244°C; IR (KBr, ν cm^{-1}): 1658 (C=O) and 3062 (NH); ^{1}H NMR (DMSO-d_6) δ *ppm*: 4.94, 5.01 (2s, 2H, CH_2), 6.88 (d, 0.5 H, Ar-H, J = 8.4 Hz), 6.99 (d, 0.5 H, Ar-H, J = 8.4 Hz), 7.24-7.42 (m, 7 H, Ar-H), 7.45 (d, 0.5 H, Ar-H, J = 8.4 Hz), 7.48 (dd, 0.5 H, Ar-H, J = 8.4, 2.4 Hz), 7.53 (d, 0.5 H, Ar-H, J = 8.4 Hz), 7.66 (s, 0.5 H, Ar-H), 7.72 (s, 0.5 H, Ar-H), 7.84-7.91 (m, 2 H, Ar-H), 8.55 (s, 0.5 H, Ar-H), 12.22, 13.21 (2s, 1H, NH, D_2O exchangeable); ^{13}C NMR (DMSO-d_6) δ *ppm*: 169.45, 164.18, 156.13, 144.98, 142.71, 137.63, 136.59, 135.42, 131.80, 129.98, 129.19, 128.05, 127.49, 126.79, 122.95, 122.83, 120.14, 112.38, 44.12; Anal. Calcd. for $C_{24}H_{16}BrClN_4OS$: C, 55.03; H, 3.08; N, 10.70, found C, 54.88; H, 3.09; N, 10.78.

1-Benzyl-5-bromo-3-(2-(4-methyl-5-(phenyldiazenyl)thiazol-2-yl)hydrazono)indolin-2-one (12a).

Red powder (yield 75%), m.p. > 300°C; IR (KBr, ν cm^{-1}): 1662 (C=O) and 3059 (NH); ^{1}H NMR (DMSO-d_6) δ *ppm*: 2.66 (s, 3H, CH_3), 4.95 (s, 2H, CH_2), 6.95 (d, 1 H, Ar-H, J = 8.4 Hz), 7.26-7.35 (m, 9 H, Ar-H), 7.49 (d, 1 H, Ar-H, J = 8.4 Hz), 7.74 (brs, 1 H, Ar-H), (d, 1 H, Ar-H, J = 8.4 Hz), 12.21 (s, 1H, NH); ^{13}C NMR (DMSO-d_6) δ *ppm*: 18.99, 43.03, 112.73, 113.08, 115.43, 122.27, 123.79, 127.82, 128.09, 129.16, 129.48, 130.00, 131.45, 133.42, 135.89, 142.00, 160.89, 179.23; MS *m/z* [%]: 531.91 [M^+, 12.15]; Anal. Calcd. for $C_{25}H_{19}BrN_6OS$: C, 56.50; H, 3.60; N, 15.81, found C, 56.69; H, 3.58; N, 15.73.

1-Benzyl-5-bromo-3-(2-(4-methyl-5-((4-nitrophenyl)diazenyl)thiazol-2-yl)hydrazono)indolin-2-one (12b).

Yellow powder (yield 81%), m.p. > 300°C °C; IR (KBr, ν cm^{-1}): 1651 (C=O) and 3186 (NH); ^1H NMR (DMSO-d_6) δ *ppm*: 2.67 (s, 3H, CH_3), 4.91 (s, 2H, CH_2), 6.92 (d, 1 H, Ar-H, J = 9.0 Hz), 7.30-7.34 (m, 7 H, Ar-H), 7.50 (d, 2 H, Ar-H, J = 8.0 Hz), 7.66 (s, 1 H Ar-H), 8.21 (d, 2 H, Ar-H, J = 8.0 Hz), 12.21 (s, 1H, NH); ^{13}C NMR (DMSO-d_6) δ *ppm*: 19.01, 57.14, 112.75, 115.43, 122.29, 123.88, 124.55, 127.61, 127.63, 127.76, 127.88, 128.09, 129.16, 130.01, 133.24, 135.91, 142.02, 160.90, 179.50; MS *m/z* [%]: 576.91 [M^+, 11.18]; Anal. Calcd. for $C_{25}H_{18}BrN_7O_3S$: C, 52.09; H, 3.15; N, 17.01, found C, 51.87; H, 3.16; N, 17.09.

1-Benzyl-5-bromo-3-(2-(5-((4-fluorophenyl)diazenyl)-4-methylthiazol-2-yl)hydrazono)indolin-2-one (12c).

Red powder (yield 70%), m.p. > 300°C °C; ^1H NMR (DMSO-d_6) δ *ppm*: 2.65 (s, 3H, CH_3), 4.95 (s, 2H, CH_2), 6.95 (d, 1 H, Ar-H, J = 8.0 Hz), 7.25-7.33 (m, 7 H, Ar-H), 7.49 (d, 2 H, Ar-H, J = 8.0 Hz), 7.93 (s, 1 H, Ar-H), 8.86 (s, 1 H, Ar-H), 12.22 (s, 1H, NH); ^{13}C NMR (DMSO-d_6) δ *ppm*: 19.00, 43.03, 112.74, 115.43, 122.28, 123.79, 127.63, 127.76, 127.83, 128.09, 129.16, 130.00, 133.41, 135.90, 142.02, 160.90, 179.23; Anal. Calcd. for $C_{25}H_{18}BrFN_6OS$: C, 54.65; H, 3.30; N, 15.30, found C, 54.82; H, 3.29; N, 15.24.

1-Benzyl-5-bromo-3-(2-(5-((4-chlorophenyl)diazenyl)-4-methylthiazol-2-yl)hydrazono)indolin-2-one (12d).

Red powder (yield 72%), m.p. > 300°C °C; ^1H NMR (DMSO-d_6) δ *ppm*: 2.66 (s, 3H, CH_3), 4.97 (s, 2H, CH_2), 6.95 (d, 1 H, Ar-H, J = 9.0 Hz), 7.25 (t, 1 H, Ar-H, J = 9.0 Hz), 7.31-7.34 (m, 6 H, Ar-H), 7.51-7.55 (m, 3 H, Ar-H), 7.71-7.73 (m, 2 H, Ar-H); ^{13}C NMR (DMSO-d_6) δ *ppm*: 19.00, 56.48, 112.73, 113.08, 115.44, 122.27, 123.80, 127.60, 127.83, 128.10, 129.16, 130.01, 131.46, 133.42, 135.98, 142.00, 153.85, 160.90, 179.24; Anal. Calcd. for $C_{25}H_{18}BrClN_6OS$: C, 53.06; H, 3.21; N, 14.85, found C, 52.91; H, 3.22; N, 14.94.

4-((2-(2-(1-Benzyl-5-bromo-2-oxoindolin-3-ylidene)hydrazinyl)-4-methylthiazol-5-yl)diazenyl)benzenesulfonamide (12e).

Red powder (yield 64%), m.p. > 300°C °C; IR (KBr, ν cm^{-1}): ^{1}H NMR (DMSO-d_6) δ *ppm*: 2.68 (s, 3H, CH_3), 4.96 (s, 2H, CH_2), 6.96 (d, 1 H, Ar-H, J = 9.0 Hz), 7.26-7.36 (m, 9 H, Ar-H), 7.50 (dd, 1 H, Ar-H, J = 9.0, 2.0 Hz), 7.93 (d, 1 H, Ar-H, J = 2.0 Hz), 8.89 (s, 1H, Ar-H), 9.17 (s, 1H, Ar-H), 12.21 (s, 1H, NH); ^{13}C NMR (DMSO-d_6) δ *ppm*: 19.00, 56.48, 112.74, 114.81, 115.44, 115.91, 122.27, 123.80, 125.51, 127.83, 128.10, 129.16, 129.94, 130.01, 133.43, 135.89, 142.01, 146.57, 157.47, 158.69, 160.90, 179.23; Anal. Calcd. for $C_{25}H_{20}BrN_7O_3S_2$: C, 49.18; H, 3.30; N, 16.06, found C, 48.99; H, 3.32; N, 16.15.

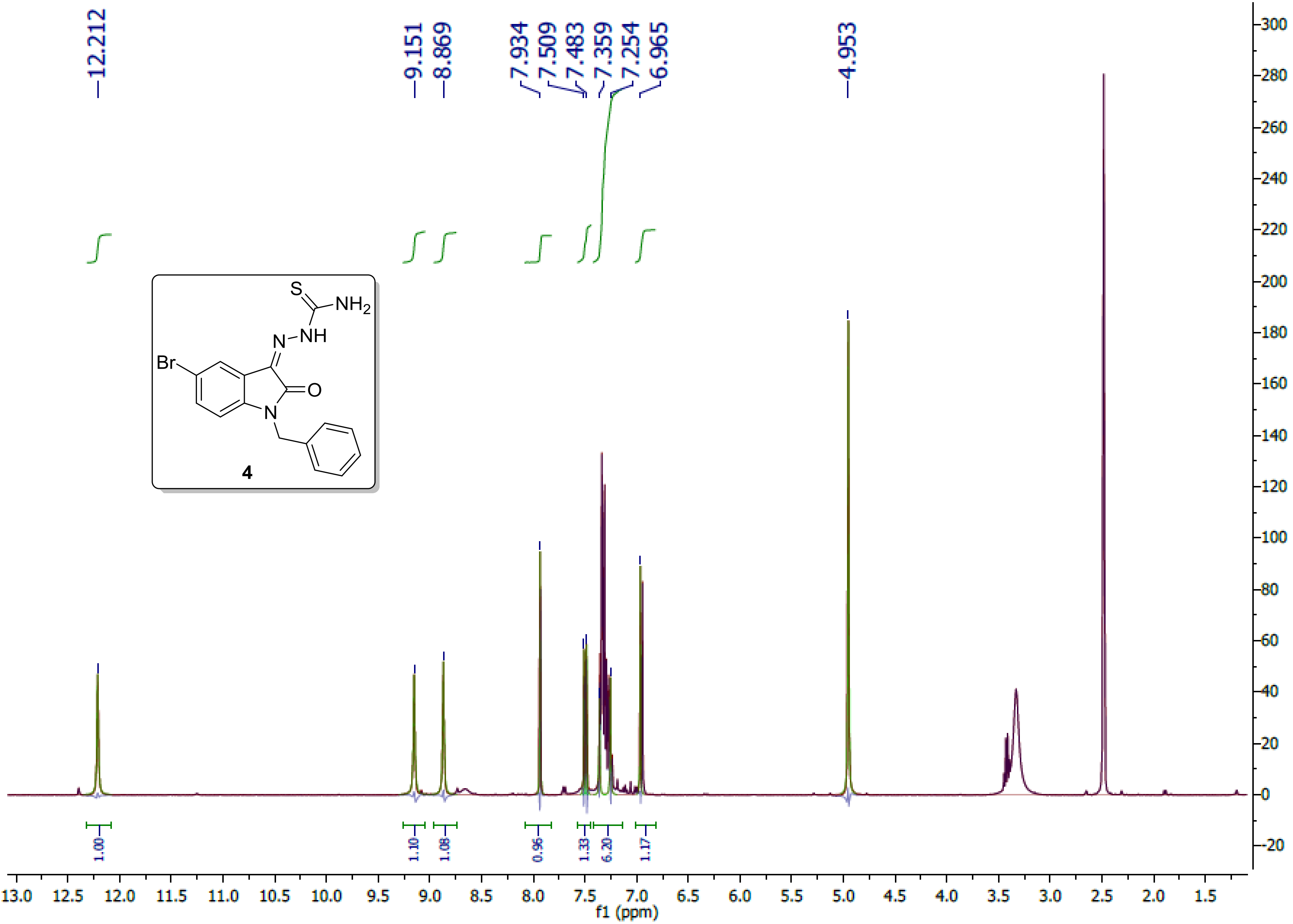

12.212
9.151
8.869
7.934
7.509
7.483
7.359
7.254
6.965
4.953
S
NH2
N
NH
Br
O
N
4
1.00
1.10
1.08
0.96
1.33
6.20
1.17
f1 (ppm)

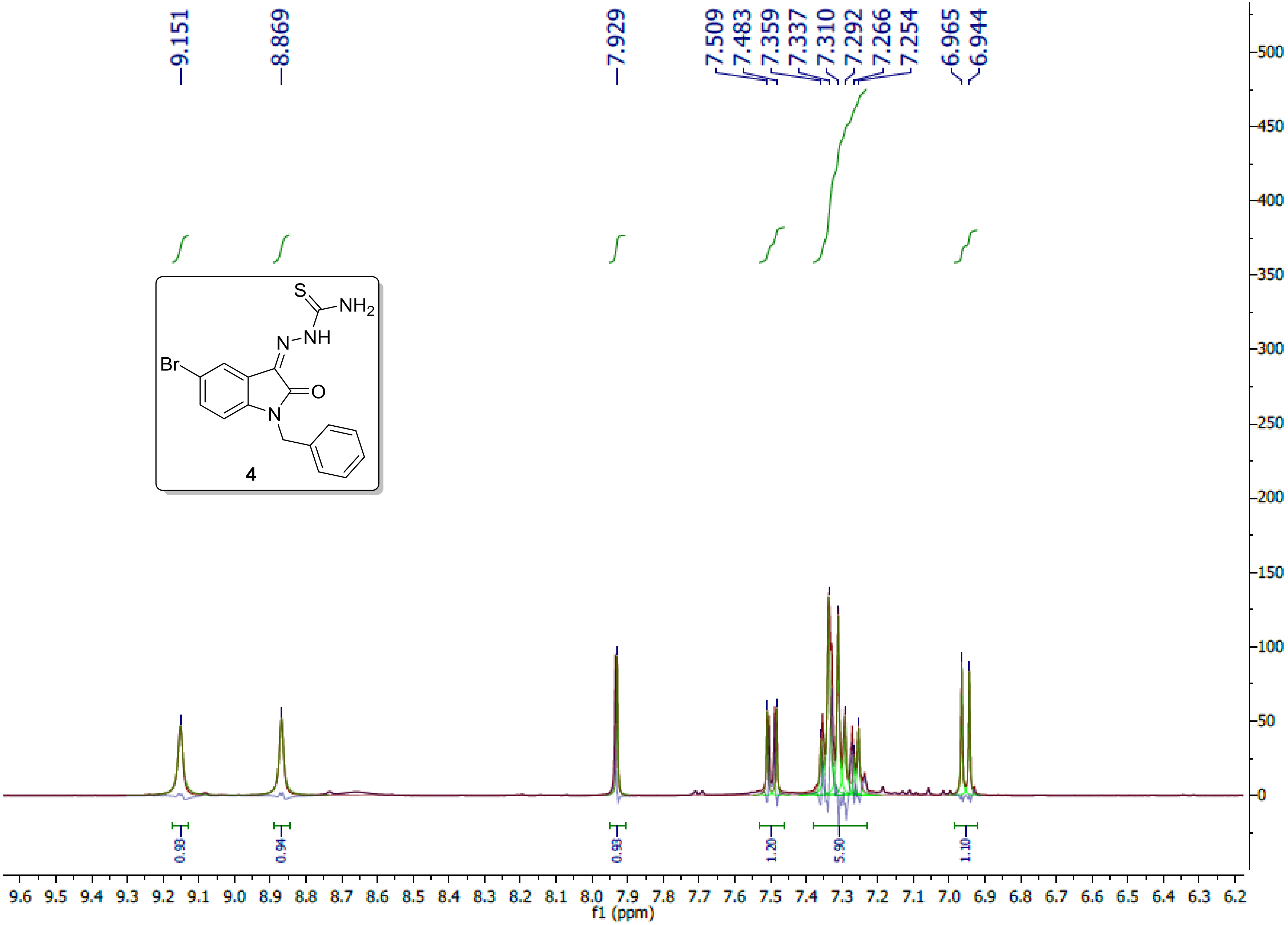
9.151
8.869
7.929
7.509
7.483
7.359
7.337
7.310
7.292
7.266
7.254
6.965
6.944
S
NH2
N
NH
Br
O
N
4
0.93
0.94
0.93
1.20
5.90
1.10
9.6 9.5 9.4 9.3 9.2 9.1 9.0 8.9 8.8 8.7 8.6 8.5 8.4 8.3 8.2 8.1 8.0 7.9 7.8 7.7 7.6 7.5 7.4 7.3 7.2 7.1 7.0 6.9 6.8 6.7 6.6 6.5 6.4 6.3 6.2
f1 (ppm)
500
450
400
350
300
250
200
150
100
50
0

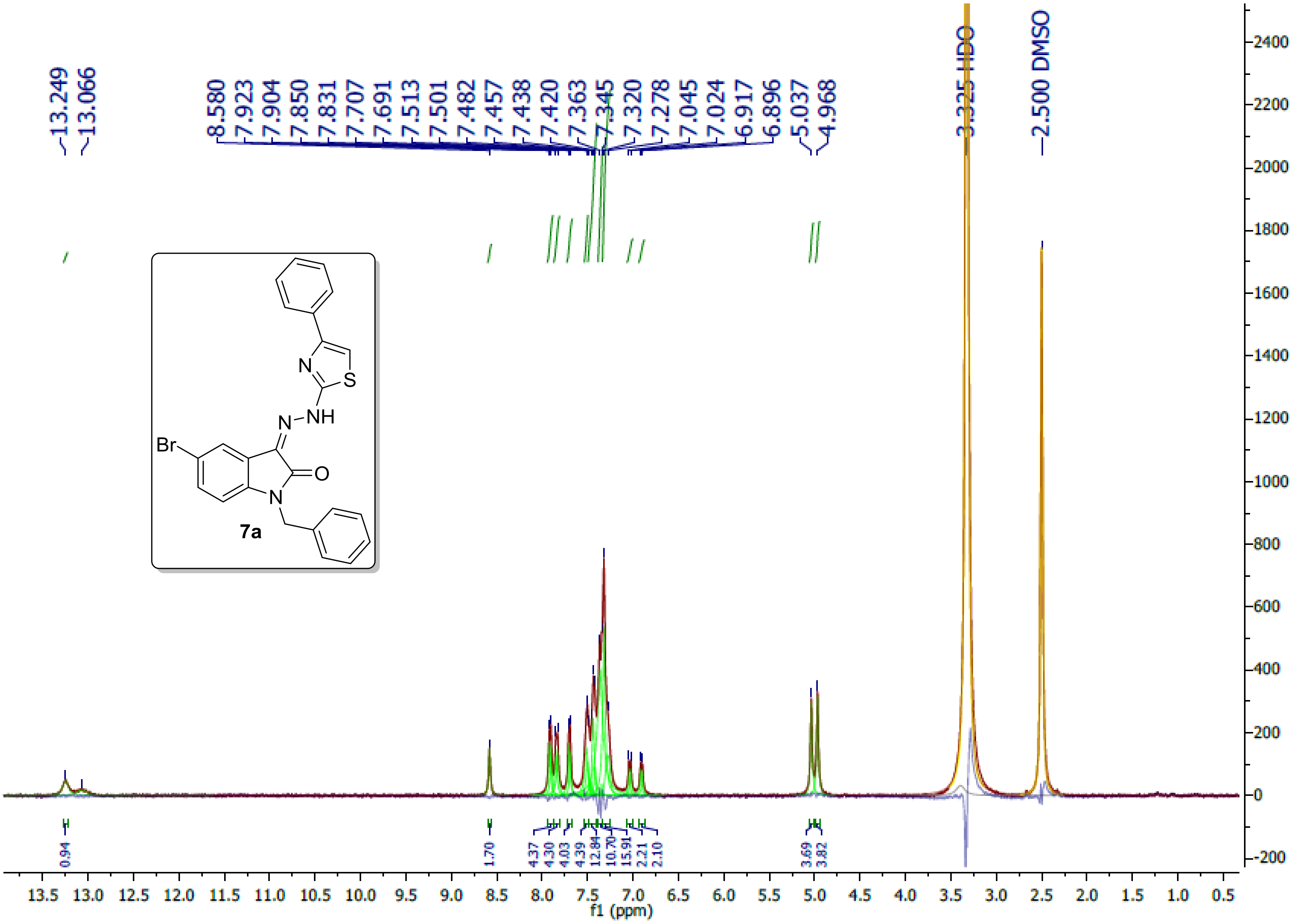

13.249
13.066
8.580
7.923
7.904
7.850
7.831
7.707
7.691
7.513
7.501
7.482
7.457
7.438
7.420
7.363
7.345
7.320
7.278
7.045
7.024
6.917
6.896
5.037
4.968
3.325 HDO
2.500 DMSO
Br
N
O
N
NH
N
S
7a
0.94
1.70
4.37
4.30
4.03
4.39
12.84
10.70
15.91
2.21
2.10
3.69
3.82
f1 (ppm)

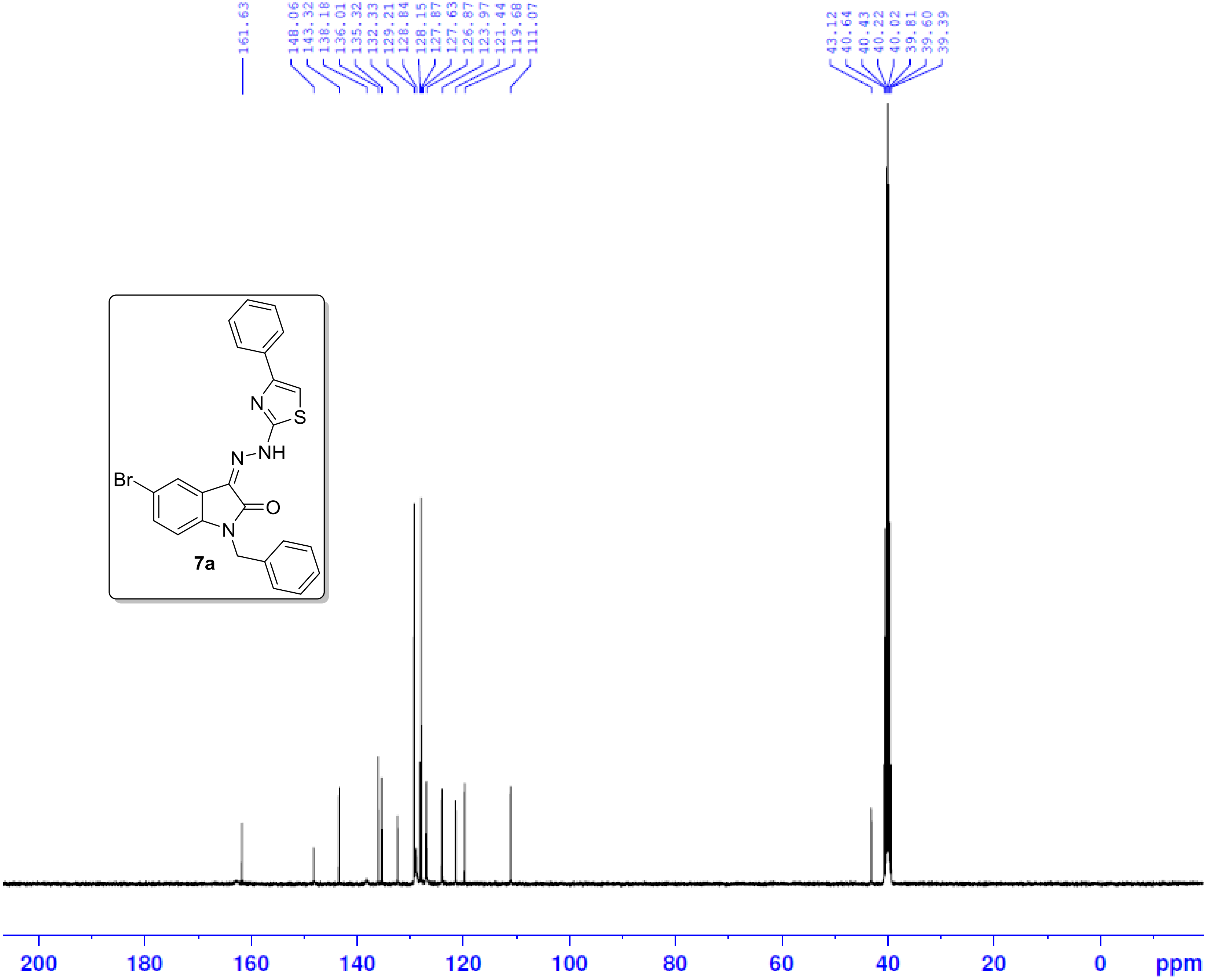
161.63
148.06
143.32
138.18
136.01
135.32
132.33
129.21
128.84
128.15
127.87
127.63
126.87
123.97
121.44
119.68
111.07
43.12
40.64
40.43
40.22
40.02
39.81
39.60
39.39
Br
N
S
N
NH
O
N
7a
200
180
160
140
120
100
80
60
40
20
0
ppm

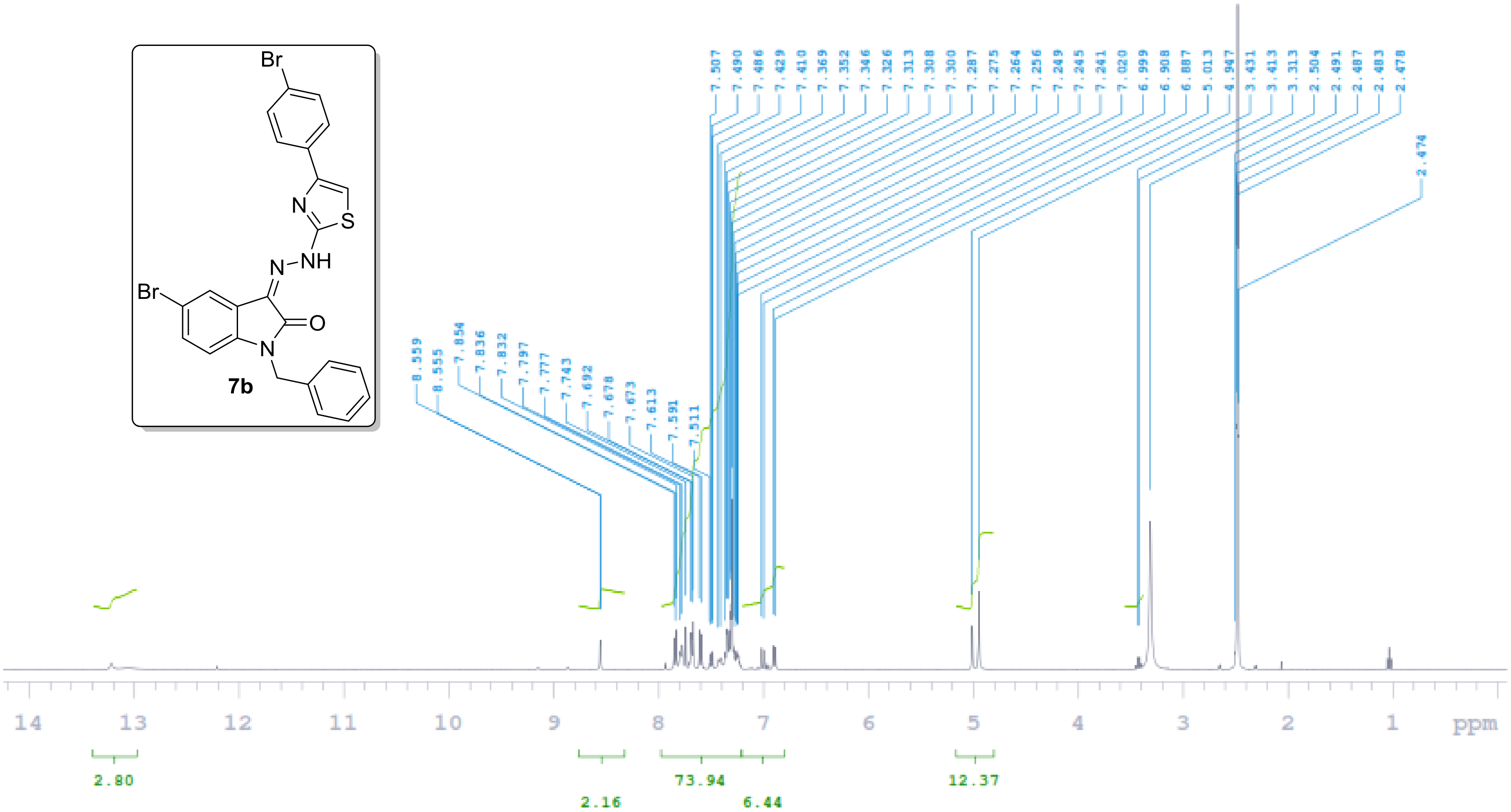

Br
N
S
N
NH
Br
O
N
7b
8.559
8.555
7.854
7.836
7.832
7.797
7.777
7.743
7.692
7.678
7.673
7.613
7.591
7.511
7.507
7.490
7.486
7.429
7.410
7.369
7.352
7.346
7.326
7.313
7.308
7.300
7.287
7.275
7.264
7.256
7.249
7.245
7.241
7.020
6.999
6.908
6.887
5.013
4.947
3.431
3.413
3.313
2.504
2.491
2.487
2.483
2.478
2.474
14 13 12 11 10 9 8 7 6 5 4 3 2 1 ppm
2.80
2.16
73.94
6.44
12.37

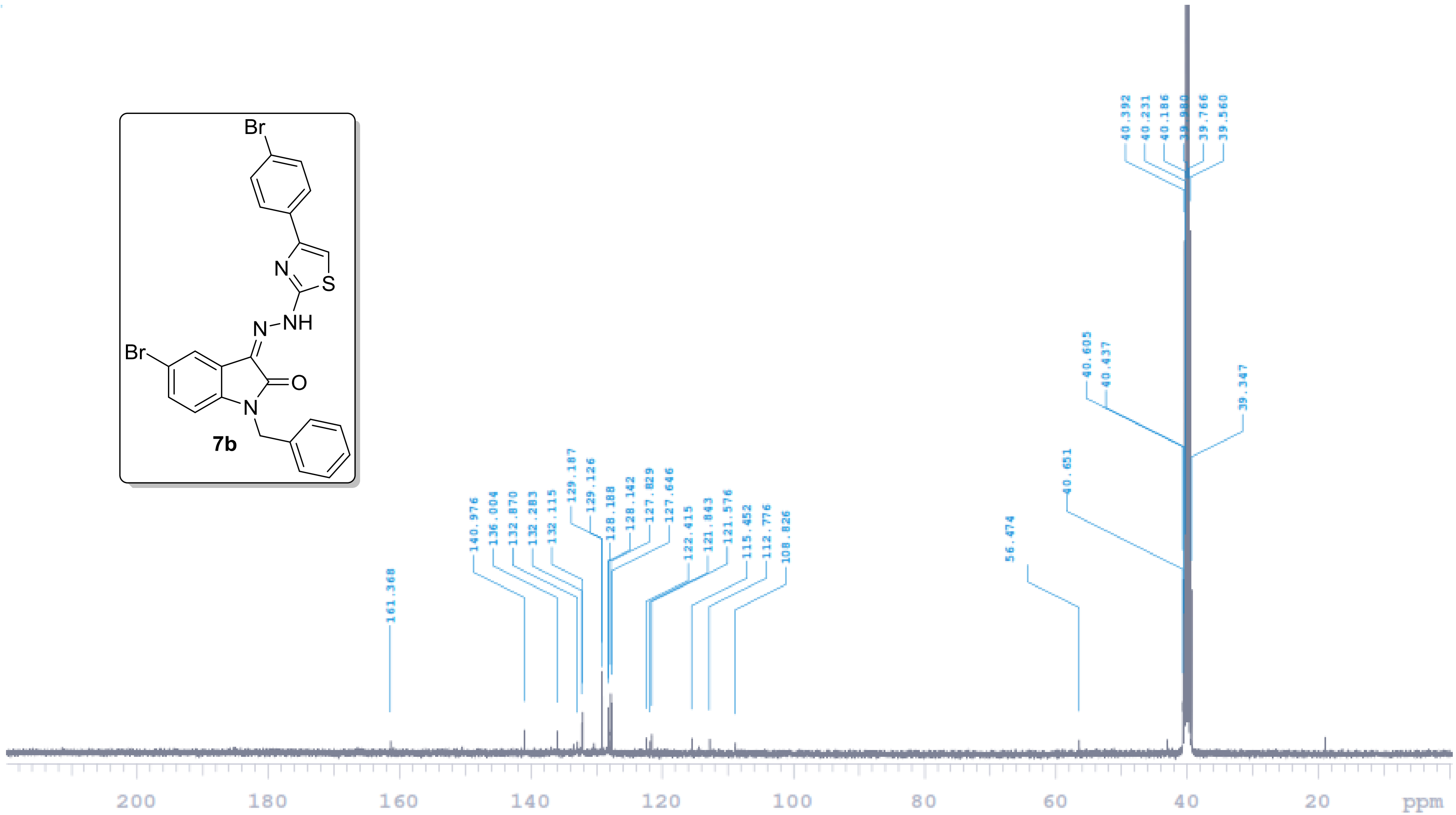
Br
N
S
NH
N
Br
O
N
7b
161.368
140.976
136.004
132.870
132.283
132.115
129.187
129.126
128.188
128.142
127.829
127.646
122.415
121.843
121.576
115.452
112.776
108.826
56.474
40.651
40.605
40.437
40.392
40.231
40.186
39.980
39.766
39.560
39.347
200
180
160
140
120
100
80
60
40
20
ppm

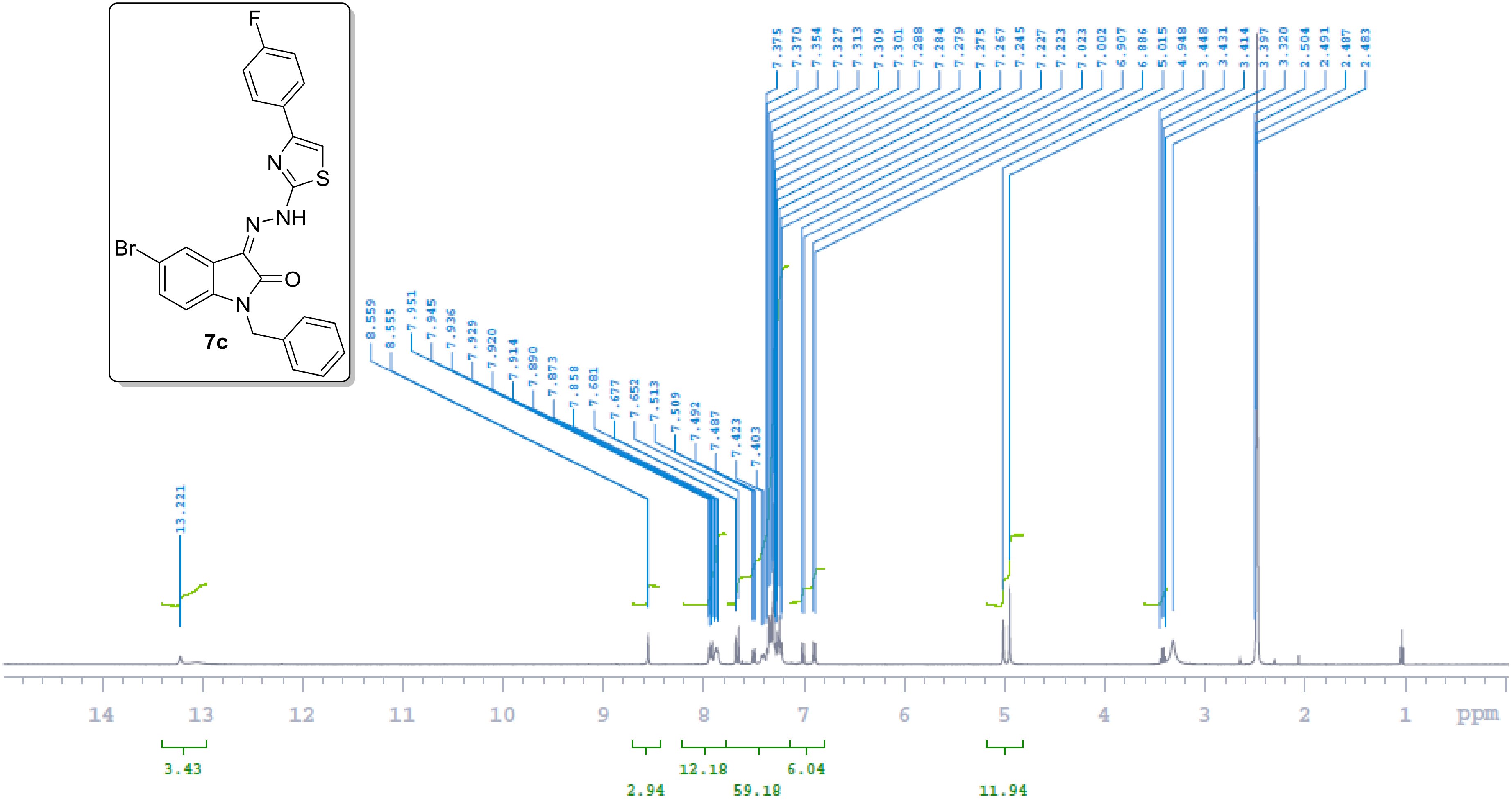
F
N
S
N
NH
Br
O
N
7c
13.221
8.559
8.555
7.951
7.945
7.936
7.929
7.920
7.914
7.890
7.873
7.858
7.681
7.677
7.552
7.513
7.509
7.492
7.487
7.423
7.403
7.375
7.370
7.354
7.327
7.313
7.309
7.301
7.288
7.284
7.279
7.275
7.267
7.245
7.227
7.223
7.023
7.002
6.907
6.886
5.015
4.948
3.448
3.431
3.414
3.397
3.320
2.504
2.491
2.487
2.483
14
13
12
11
10
9
8
7
6
5
4
3
2
1
ppm
3.43
2.94
12.18
59.18
6.04
11.94

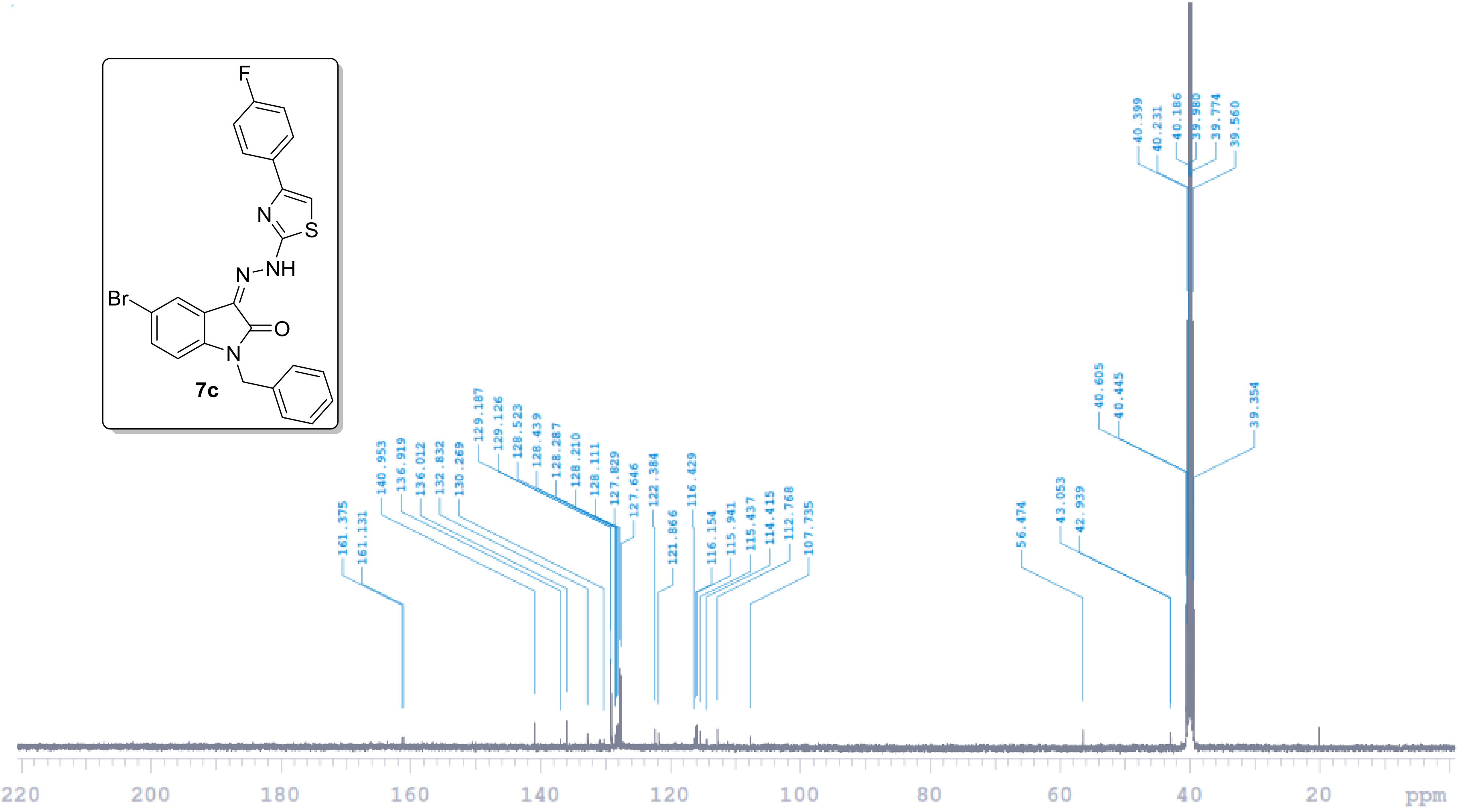
F
N
S
N
NH
Br
O
N
7c
161.375
161.131
140.953
136.919
136.012
132.832
130.269
129.187
129.126
128.523
128.439
128.287
128.210
128.111
127.829
127.646
122.384
121.866
116.429
116.154
115.941
115.437
114.415
112.768
107.735
56.474
43.053
42.939
40.605
40.445
40.399
40.231
40.186
39.980
39.774
39.560
39.354
220
200
180
160
140
120
100
80
60
40
20
ppm

Dr_Rizk_Ayad-R6d

Sample Name **Dr_Rizk_Ayad-R6d**
Date collected **2016-08-06**

Pulse sequence **PROTON**
Solvent **dmso**

Temperature **25**
Spectrometer **nmr400-mercury400**

Study owner **vnmr1**
Operator **vnmr1**

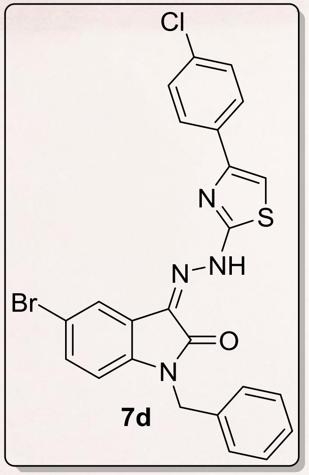

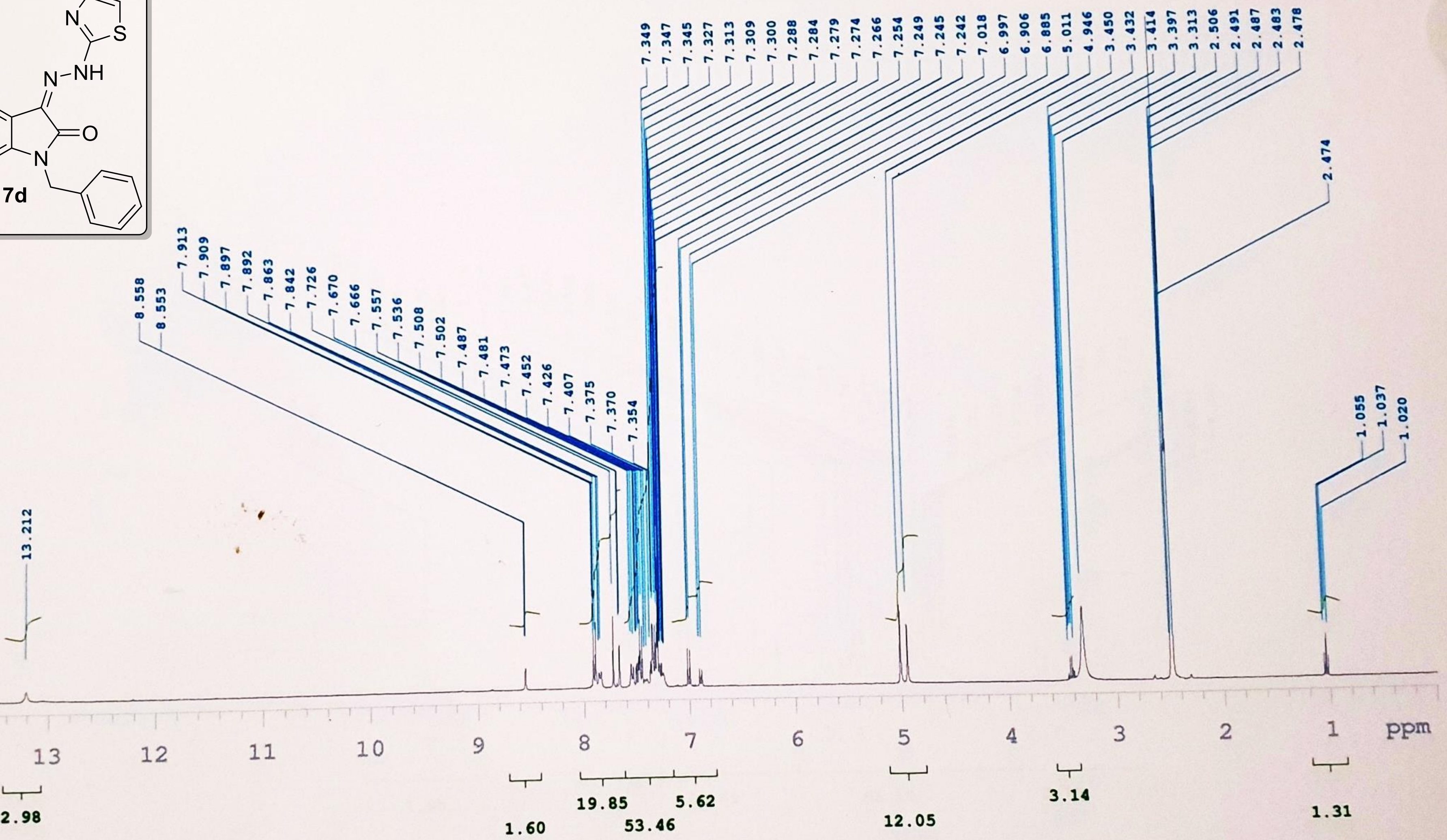

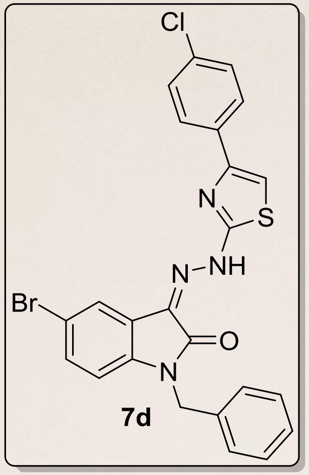

Dr_Rizk_Ayad-R6d

Sample Name	Dr_Rizk_Ayad-R6d	Pulse sequence	PROTON	Temperature	25	Study owner	vnmr1
Date collected	2016-08-06	Solvent	dmso	Spectrometer	nmr400-mercury400	Operator	vnmr1

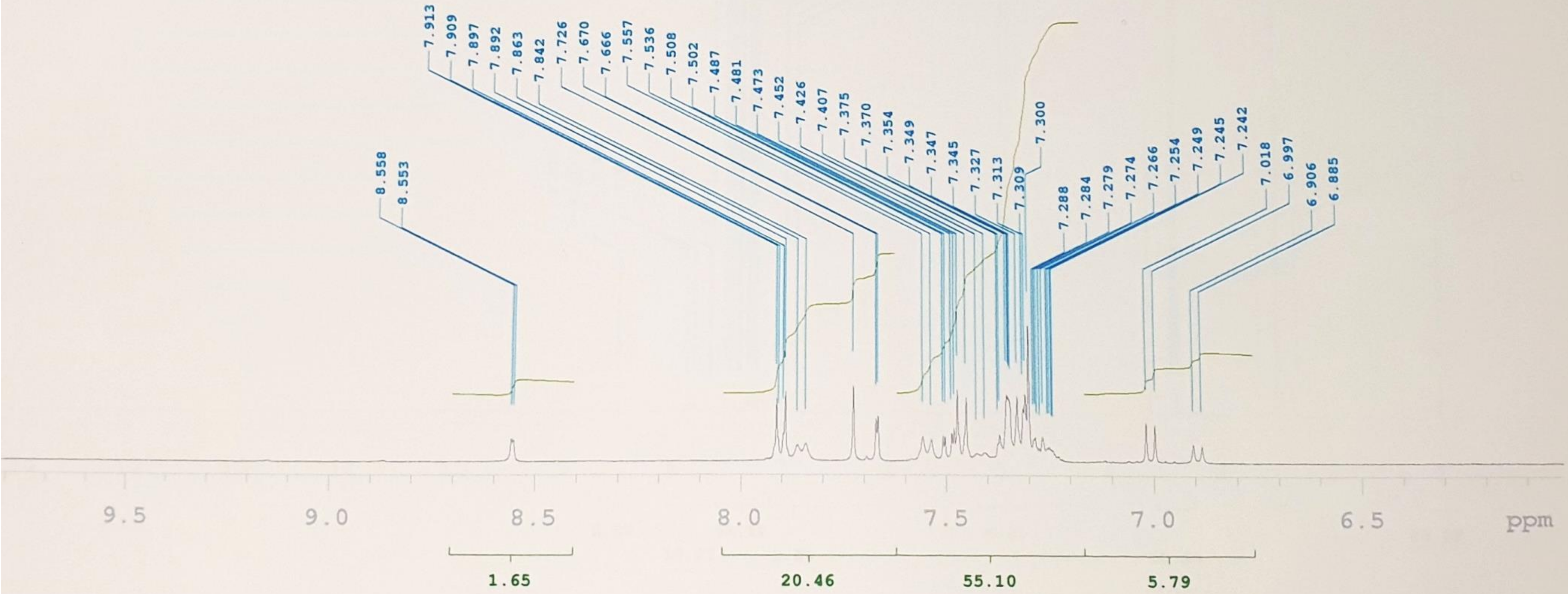

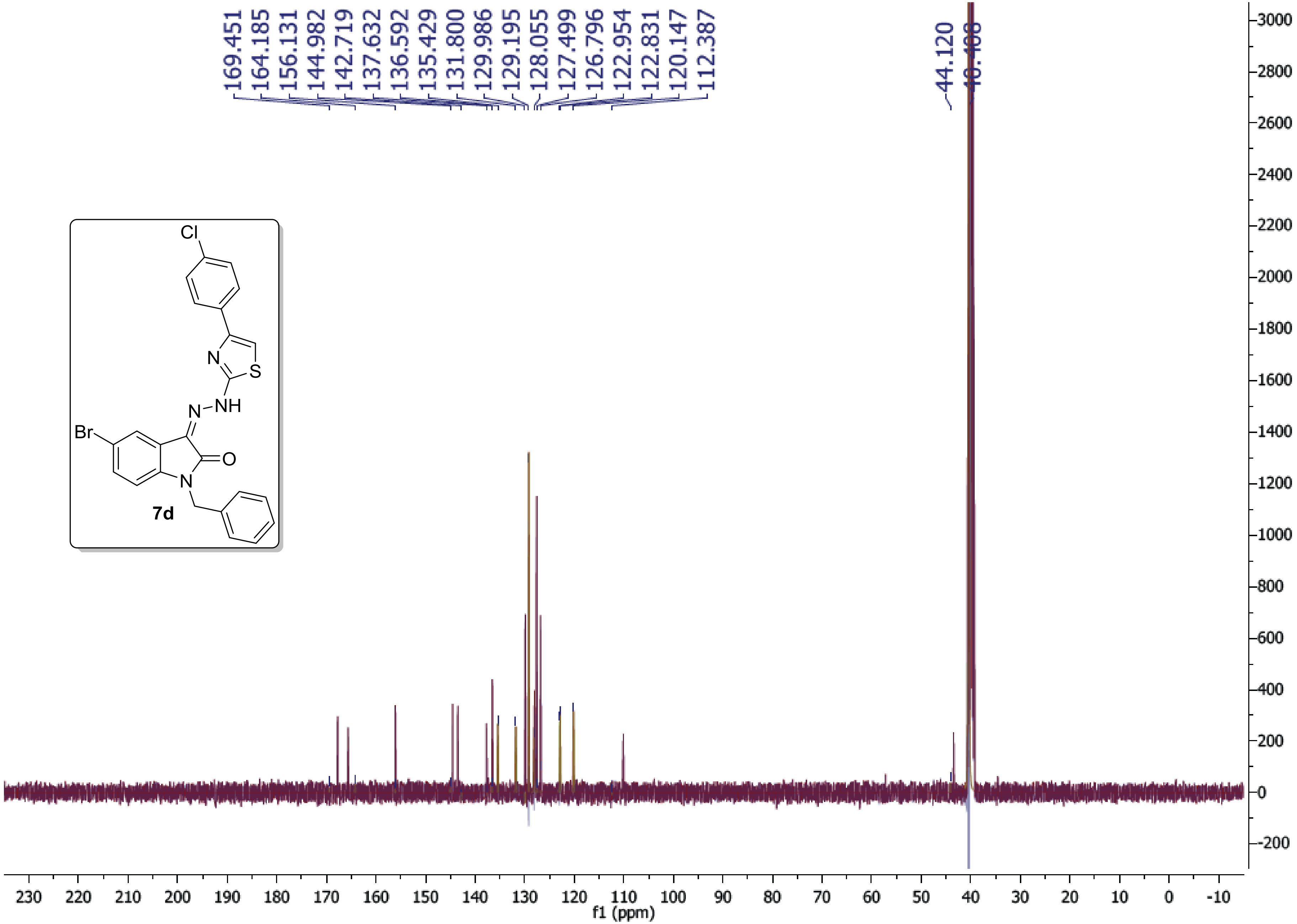
169.451
164.185
156.131
144.982
142.719
137.632
136.592
135.429
131.800
129.986
129.195
128.055
127.499
126.796
122.954
122.831
120.147
112.387
44.120
40.408
Cl
N
S
N
NH
Br
O
N
7d
f1 (ppm)

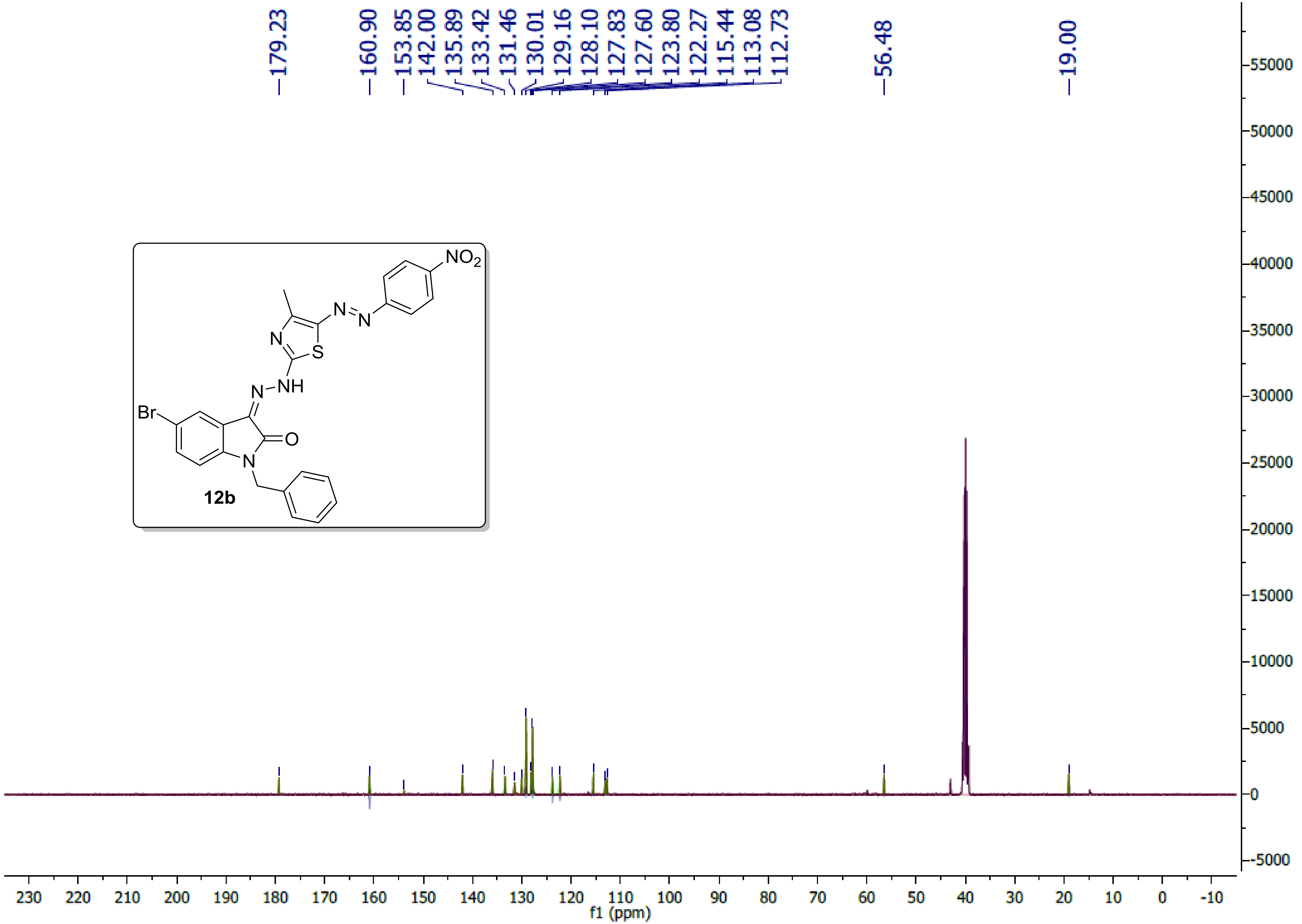

179.23
160.90
153.85
142.00
135.89
133.42
131.46
130.01
129.16
128.10
127.83
127.60
123.80
122.27
115.44
113.08
112.73
56.48
19.00
NO2
N
N
N
S
N
NH
Br
O
N
12b
f1 (ppm)

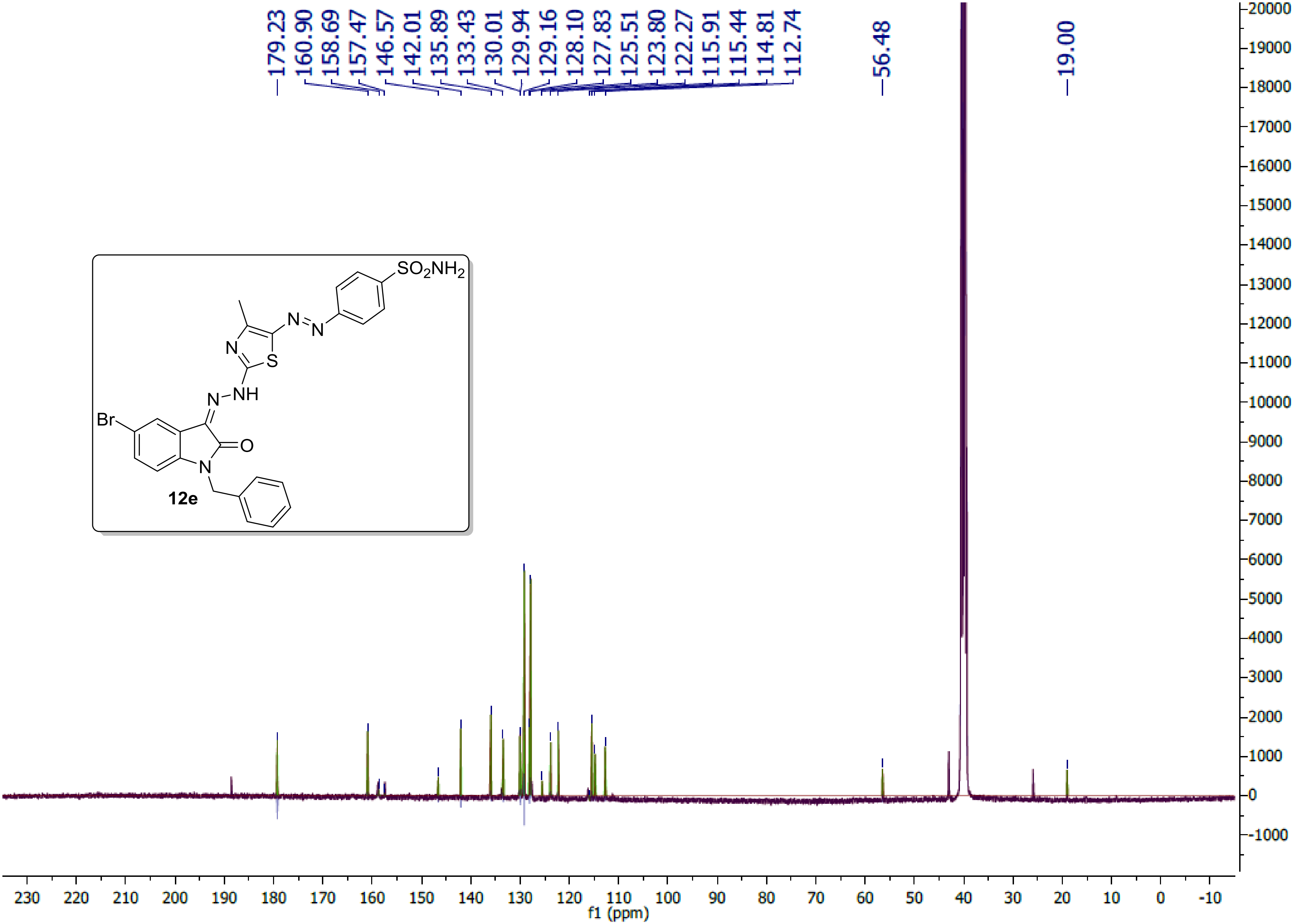

179.23
160.90
158.69
157.47
146.57
142.01
135.89
133.43
130.01
129.94
129.16
128.10
127.83
125.51
123.80
122.27
115.91
115.44
114.81
112.74
56.48
19.00
SO2NH2
N
N
N
S
N
NH
Br
O
N
12e
f1 (ppm)
